# Image Reconstruction in Light-Sheet Microscopy: Spatially Varying Deconvolution and Mixed Noise

**DOI:** 10.1007/s10851-022-01100-3

**Published:** 2022-06-14

**Authors:** Bogdan Toader, Jérôme Boulanger, Yury Korolev, Martin O. Lenz, James Manton, Carola-Bibiane Schönlieb, Leila Mureşan

**Affiliations:** 1 Cambridge Advanced Imaging Centre, University of Cambridge, Anatomy School, Downing Street, Cambridge CB2 3DY, UK; 2 MRC Laboratory of Molecular Biology, Francis Crick Avenue, Cambridge CB2 0QH, UK; 3 Department of Applied Mathematics and Theoretical Physics, University of Cambridge, Wilberforce Road, Cambridge CB3 0WA, UK; 4 Department of Physiology, Development and Neuroscience, University of Cambridge, Downing Street, Cambridge CB2 3DY, UK; 5 Sainsbury Laboratory, University of Cambridge, 47 Bateman Street, Cambridge CB2 1LR, UK

**Keywords:** Deconvolution, Light-sheet microscopy, Poisson and Gaussian noise, Primal–dual hybrid gradient, Numerical methods

## Abstract

We study the problem of deconvolution for light-sheet microscopy, where the data is corrupted by spatially varying blur and a combination of Poisson and Gaussian noise. The spatial variation of the point spread function of a light-sheet microscope is determined by the interaction between the excitation sheet and the detection objective PSF. We introduce a model of the image formation process that incorporates this interaction and we formulate a variational model that accounts for the combination of Poisson and Gaussian noise through a data fidelity term consisting of the infimal convolution of the single noise fidelities, first introduced in L. Calatroni et al. (SIAM J Imaging Sci 10(3):1196–1233, 2017). We establish convergence rates and a discrepancy principle for the infimal convolution fidelity and the inverse problem is solved by applying the primal–dual hybrid gradient (PDHG) algorithm in a novel way. Numerical experiments performed on simulated and real data show superior reconstruction results in comparison with other methods.

## 1 Introduction

**Fig. 1:**
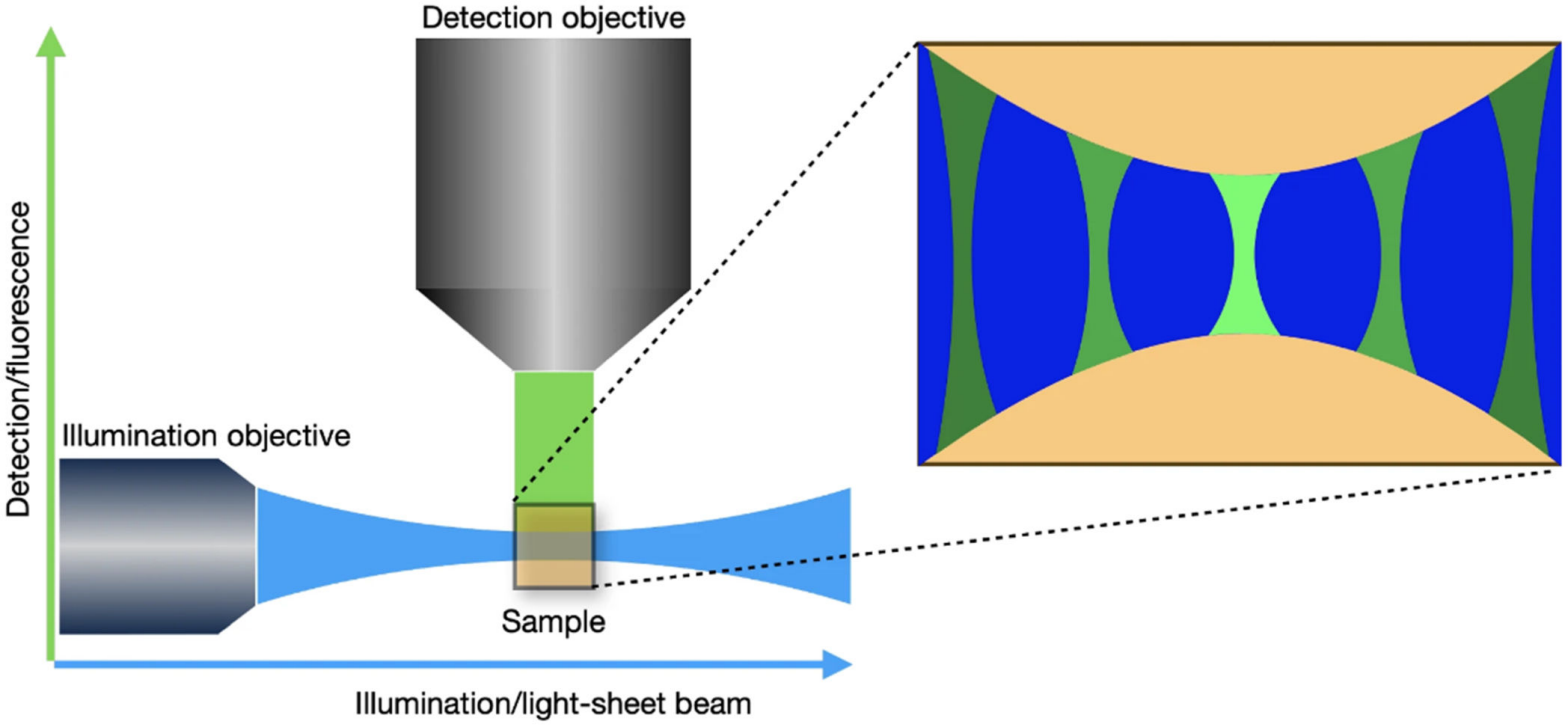
Schematic of a light-sheet microscope, showing the illumination and the detection directions. The interaction of the light-sheet with the detection PSF leads to a spatially varying overall PSF and decreasing of the pixel intensities away from the centre in the horizontal direction

Light-sheet microscopy is a fluorescence microscopy technique that enables volumetric imaging of biological samples at high frame rate with better sectioning and lower photo-toxicity in comparison with other fluorescent techniques. This is achieved by illuminating a thin slice of the sample using a sheet of light and detecting the emitted fluorescence from this plane with another objective perpendicular to the plane of the sheet. A schematic representation of a light-sheet microscope is shown in Fig. 1. Other microscopy techniques present certain disadvantages. For example, wide-field microscopy [1] illuminates the whole sample using a single objective and achieves only very limited sectioning, while confocal microscopy [1] allows improved sectioning by utilising a pinhole to discard out-of-focus light, at the cost of higher photo-toxicity and reduced frame rate. Light-sheet microscopy avoids these downsides by only selectively illuminating the slice of the sample being imaged. In this way, less photo-toxicity damage is induced and, therefore, imaging of living samples over a longer period of time is possible. The combination of lower photo-toxicity, better sectioning capabilities and faster image acquisition led to light-sheet microscopy being recognised as “Method of the Year” by Nature Methods in 2014 [2].

The focus of the present manuscript is on deconvolution techniques for light-sheet microscopy data. In this context, deconvolution refers to the computational method of reversing the effect of blurring in the image acquisition process due to the point spread function (PSF) of the microscope [3–5]. Specifically, the PSF of an imaging system represents its response to a point object. In general, knowledge of the PSF can be modelled mathematically and calibrated using bead data (samples containing small spheres of known dimensions), and then used in the formulation of a forward model of the image formation, which can then be inverted, for example using optimisation methods, to reconstruct the original, deblurred object [6].

However, in the case of light-sheet microscopy, simply knowing or estimating the PSF of the detection objective is not sufficient, since the overall response of the system to a point source is also influenced by the excitation light-sheet used to illuminate the slice. The overall PSF could be approximated by the detection PSF in the region where the illumination sheet is focused. However, the detection PSF becomes more distorted and loses intensity away from the focus of the excitation light-sheet, an effect illustrated in Fig. 1, so this approach is not accurate. Therefore, we address this problem as a case of spatially varying deconvolution [7,8], where the variation of the system’s overall PSF is determined by the interaction between the detection PSF and the light-sheet. We note that, in general, the detection PSF itself can be spatially varying due to optical aberrations in the sample, a problem that is not specific to light-sheet microscopy. We do not address this source of variability in this work, although such a spatially varying detection PSF could in principle be incorporated in our method.

**Fig. 2:**
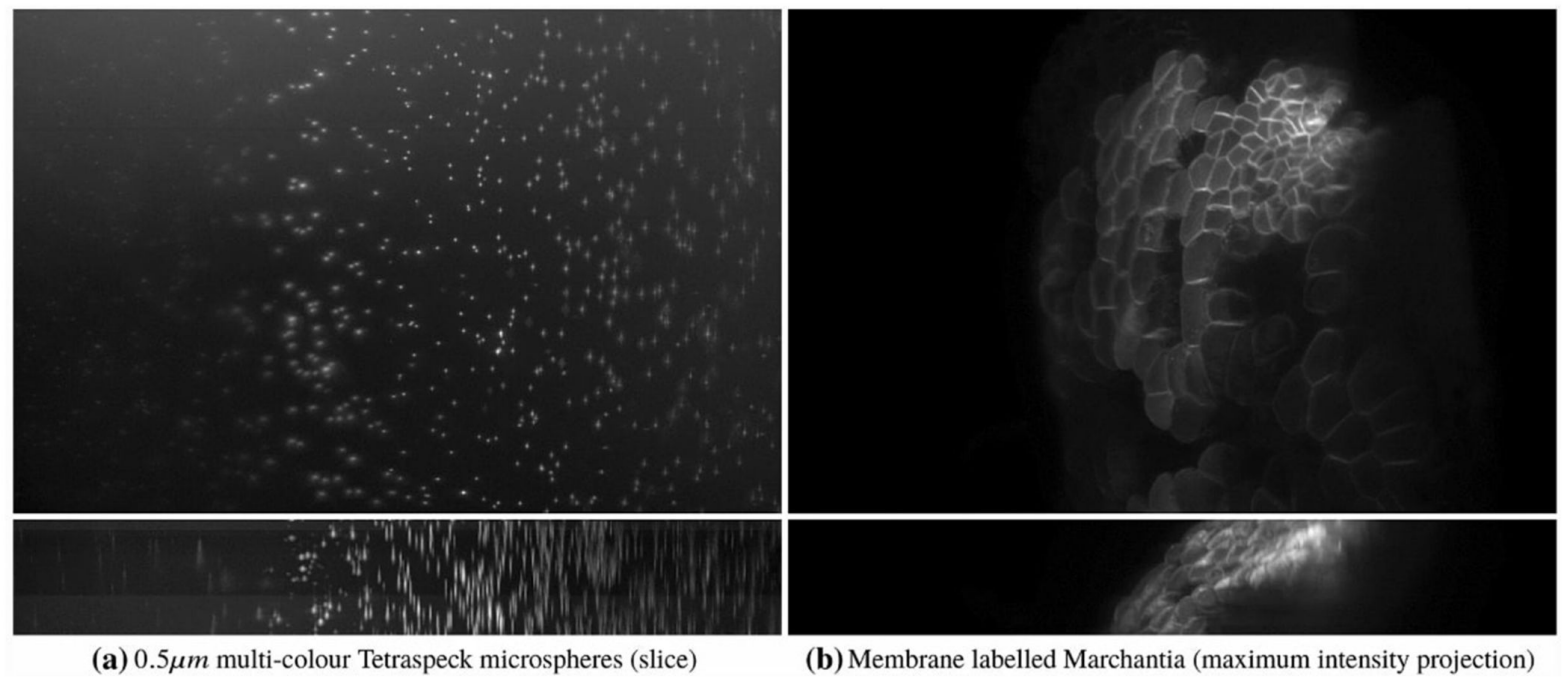
Examples of light-sheet microscopy data of dimensions 665.6 μm × 665.6 μm: beads in **a** and Marchantia thallus in **b**. We show for both samples maximum intensity projections on the *x*−*y* plane in the top row and on the *x* − *z* plane in the bottom row, with the bead intensity shown in log scale for increased contrast. The effect of the light-sheet is visible along the horizontal direction (the *x* axis), as the image is sharp and has higher intensity in the centre, where the sheet is focused, while the quality of the image decreases away from the centre. The blurring effect of the light-sheet in the *z* direction is particularly noticeable in the *x* − *z* projections in the bottom row. Another source of blur observed, especially in the bead image (left) is given by optical aberrations due to the sample imaging medium. The Marchantia image has been acquired using samples from Dr. Alessandra Bonfanti and Dr. Sarah Robinson using the genetic line provided by Prof. Sebastian Schornack and Dr. Giulia Arsuffi at the Sainsbury Laboratory Cambridge University

Two examples of acquired data are shown in Fig. 2. We can see in both cases the effect of the spatially varying light-sheet: the image is sharper in the centre and blurry on the sides, with the amount of blur growing with the horizontal distance from the centre. In addition, the fluorescence intensity of imaged beads in Fig. 2a is unevenly distributed despite imaging a homogeneous sample of beads, with the centre of the image being brighter than the left and right sides. The aim of our work is to correct these effects.

### 1.1 Contribution

We propose a method for deconvolution of 3D light-sheet microscopy data that takes into account the spatially varying nature of the PSF and is scalable to the dimensions typical to biological samples imaged using light-sheet microscopy—4.86GB per 3D 16-bit stack of 2048 × 2048 × 580 voxels.

Our approach is based on a new model for image formation that describes the interaction between the light-sheet and the detection PSF which replicates the physics of the microscope. Then, we formulate an inverse problem where the forward operator is given by model of the image formation process and which takes into account the degradation of the data by both Gaussian and Poisson noise as an infimal convolution (a concept that will be defined in Sect. 3) between an L^2^ term and a Kullback–Leibler divergence term, following [9]. The proposed variational problem is solved by applying the Primal Dual Hybrid Gradient (PDHG) algorithm [6,10,11] in a novel way. Finally, we exploit the noise model to automatically tune the balance between the data fidelity and regularisation resorting to a discrepancy principle. We obtain convergence rates in a Bregman distance for the infimal convolution fidelity from [9] under a standard source condition.

In our numerical experiments, we first show how this method performs on simulated data, where the ground truth is known, then we apply our method to two examples of data from experiments: an image of fluorescent beads and a sample of Marchantia. In both cases, we see that the deconvolved images show improved contrast, while outperforming deconvolution using only the constant detection PSF.

### 1.2 Related Work

Before describing in more detail our approach to the deconvolution problem, we give a brief overview of the literature on spatially varying deconvolution in the context of microscopy and how our work relates to it.

Purely data-driven approaches estimate a spatially varying PSF in a low dimensional space (for scalability reasons) using bead images [7,8,12]. This is usually not application specific and can be included in a more general blind deconvolution framework. Similarly, the work in [13] involves writing the spatially varying PSF as a convex combination of spatially invariant PSFs. The algorithm alternates between estimating the image and estimating the PSF. In a similar vein, the authors of [14] approach the problem of blind deconvolution by defining the convolution operator using efficient matrix-vector multiplication operations. This decomposition is similar to the discrete formulation of our image formation model. These methods optimise over the (unknown) operator in addition to the unknown image. Related to these results is [15], where the authors consider the models from [12] and [14] under the assumption that the blurring operator is known and given as a sum of weighted spatially invariant operators. They exploit this structure of the operator and use a Douglas–Rachford-based splitting to solve the optimisation problem efficiently. A different data-driven approach is presented in [16], where a deep artificial neural network is used to learn the spatially varying PSF from simulated data obtained using a forward model of the microscope. While these approaches are more general than our method, we consider that using the knowledge of the image formation process in the forward model is advantageous for the reconstruction of light-sheet microscopy data.

A number of groups consider the problem of reconstruction from multiple views in the context of light-sheet microscopy. In [17], the problem of multi-view reconstruction under a spatially varying blurring operator for 3D light-sheet data is considered. They divide the image into small blocks where they perform deconvolution using spatially invariant PSFs estimated from beads (and interpolated PSFs in regions where there are no beads). In [18], the authors extend the Richardson–Lucy algorithm to the multi-view reconstruction problem in a Bayesian setting. While it allows for different PSFs for each view (estimated using beads), this work does not consider spatial variations of the PSF. While using data from multiple views improves the quality of the reconstruction, these approaches are agnostic to the physics of the microscope.

The approach taken in [19,20] involves directly measuring the spatially varying PSF in different regions of the field of view using an additional hardware module installed with the microscope, and then deconvolving the image in each region using the measured PSF. In particular, [20] employs a sophisticated tiling-based deconvolution method based on the Richardson–Lucy algorithm and a formulation similar to a convolutional neural network in order to avoid artefacts usually caused by stitching tiles deconvolved with different PSFs.

Taking an approach similar in spirit to ours, the authors of [21] model the effective PSF of a light-sheet microscope, which is then plugged into a regularised version of the Richardson–Lucy algorithm for deconvolution. However, while they model the detection PSF and the light-sheet separately, they assume the effective PSF of the microscope is spatially invariant and the point-wise product of the two PSFs. In contrast, we do not take this simplifying step in our modelling, as we consider that the relationship between the two PSFs plays in important role in the resulting blur of the image.

The work of Guo et al. [22] uses a modified Richardson–Lucy algorithm implemented on GPU to improve the speed of convergence, further improved by the use of a deep neural network, which is a promising approach.

Moreover, in [23] the authors introduce an image formation model similar to the one described in the present manuscript. However, the regions of the resulting PSF where the light-sheet is out of focus are discarded, hence approximating the overall PSF with a constant PSF and then performing deconvolution using the ADMM algorithm. In Cueva et al. [24], a mathematical model which takes into account image fusion with two-sided illumination is derived from first principles. However, it is restricted to 2D and they do not apply the method to real data.

Lastly, regarding the mixed Gaussian–Poisson noise fidelity, our method follows the infimal convolution variational approach described in [9], with the additional light-sheet blurring operator. The same inverse problem, without the blurring operator, is solved in [25] albeit using an ADMM algorithm for the minimisation.

### 1.3 Paper Structure

The paper is organised as follows. In Sect. 2, we introduce a mathematical model of the image formation process in a light-sheet microscope. This model describes how the sample is blurred by the excitation illumination together with the detection objective PSF. Optical aberrations of the system are modelled using Zernike polynomials in the detection PSF, which we discuss in Sect. 2.3. In Sect. 3, we define the mathematical setting for the deconvolution problem and we state an inverse problem using a data fidelity as an infimal convolution of the individual Gaussian and Poisson data fidelities. We discuss convergence rates and a discrepancy principle for choosing the regularisation parameter in Sect. 2.1. In Sect. 4, we describe how PDHG is applied to this inverse problem, with details of the implementation of the proximal operator and the convex conjugate of the joint Kullback–Leibler divergence. Finally, we validate our method with numerical experiments both with simulated and real data in Sect. 5, before concluding and giving a few directions for future work in Sect. 6.

## 2 Forward Model

The first contribution of the current work is a model of the image formation process in light-sheet microscopy. By modelling the excitation light-sheet and the detection PSF separately and their interaction in a way that replicates the physics of the microscope, we are able to accurately simulate the spatially varying PSF of the imaging system. We then incorporate this knowledge as the forward model in an inverse problem, which we solve to remove the noise and blur in light-sheet microscopy data. In this section, we describe the image formation process and the PSF model.

### 2.1 Image Formation Model

**Fig. 3:**
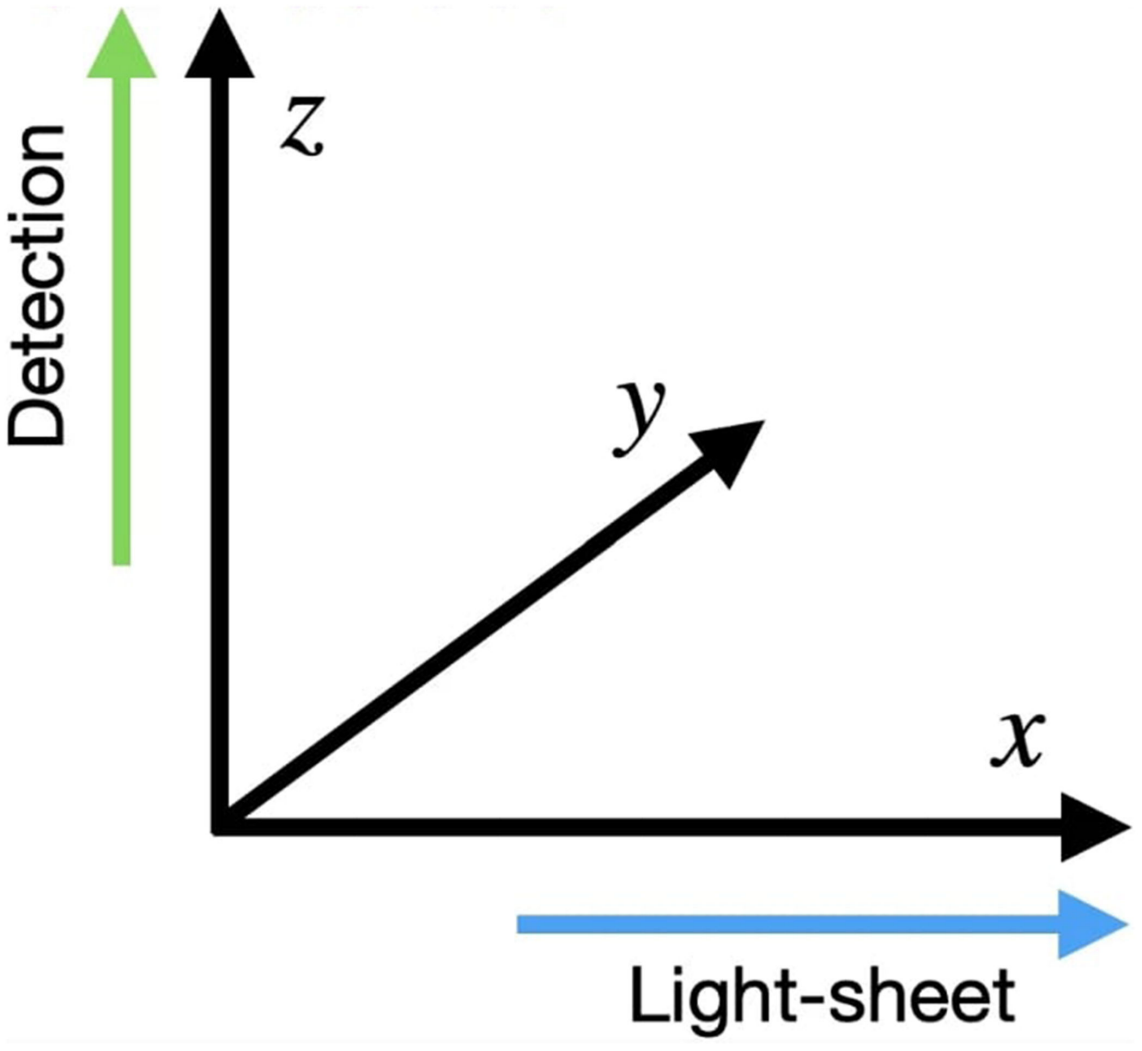
Coordinate axes showing the light-sheet beam direction along the *x* axis and the detection direction along the *z* axis

A light-sheet propagated along the *x* direction is focused by the excitation objective at an axial position *z* = *z*_0_ and the local light-sheet intensity *l* is modelled by the incoherent point spread function (PSF) of the excitation objective. The sample with local density of fluorophores *u* emits photons proportionally to the local intensity *l* of the light-sheet. These photons are then collected by a detection objective, whose action on the illuminated sample is modelled as a convolution with its PSF *h*. For clarity, see Fig. 3 for the directions of the axes. Finally, the sensor conjugated with the image plane *z*_0_ collects photons and converts them to digital values for storage. Consequently, the recorded image is corrupted by a combination of Gaussian and Poisson noise. We can see here again how the local variation of the light-sheet will result in a spatially varying blur and spatially varying illumination intensity in the captured image. This process is then repeated for each *z*_0_ to obtain the measured data *f*.

More specifically, we model *u*, *f*, *l* and *h* as functions defined on $\Omega \subset {\mathbb{R}}^{3}$, a rectangular domain of dimensions Ω_*x*_ × Ω*_y_* × Ω*_z_* (in μm) with $\Omega =\left[-\frac{\Omega_{x}}{2},\frac{\Omega_{x}}{2}\right]\times \left[-\frac{\Omega_{y}}{2},\frac{\Omega_{y}}{2}\right]\times \left[-\frac{\Omega_{z}}{2},\frac{\Omega_{z}}{2}\right]$. For the sample *u*, the light-sheet *l* and the detection objective PSF *h*, the measured data *f* is given by:

(2.1) $$\begin{array}{c} f(x,y,z)=\int \int \int \;u(s,t,w-z)l(s,t,w)h(x-s,y-t,-w)\text{d}s\text{d}t\text{d}w. \end{array}$$

The detection PSF *h* is given by

(2.2) $$\begin{array}{c} \;h(x,y,z)={\left|\iint g_{\sigma }*p_{Z}(\kappa_{x},\kappa_{y})e^{2i\pi z\sqrt{{\left(n/\lambda_{h}\right)}^{2}-\kappa_{x}^{2}-\kappa_{y}^{2}}}e^{2i\pi (\kappa_{x}x+\kappa_{y}y)}\text{d}\kappa_{x}\text{d}\kappa_{y}\right|}^{2} \end{array}$$

and the light-sheet *l* is the y-averaged beam PSF *l_beam_*:

(2.3) $$\begin{array}{c} \;l_{\mathrm{beam}}(x,y,z)={\left|\iint p_{0}(\kappa_{z},\kappa_{y})e^{2i\pi x\sqrt{{\left(n/\lambda_{l}\right)}^{2}-\kappa_{z}^{2}-\kappa_{y}^{2}}}e^{2i\pi (\kappa_{z}z+\kappa_{y}y)}\text{d}\kappa_{z}\text{d}\kappa_{y}\right|}^{2}, \end{array}$$

where *n* is the refractive index, *λ_h_*, *λ_l_* are the wave lengths corresponding to the detection objective and light-sheet beam, respectively, and *g_σ_* represents Gaussian blur. Lastly, *p_Z_* (*κ_x_*, *κ_y_*) and *p*_0_(*κ_z_, κ_y_*) are the pupil functions for the detection PSF and the light-sheet beam, respectively, both given by:

(2.4) $$p_{\varphi }\left(x,y\right)=\{\begin{array}{ll} e^{2i\pi \varphi } & \text{for}\;\sqrt{x^{2}+y^{2}}\leq NA_{i}/\lambda_{i},\\ 0 & \text{otherwise}, \end{array}$$

for their respective wave lengths, *λ_i_* = *λ_h_* or *λ_i_* = *λ_l_*, and numerical apertures, *NA_i_* = *NA_h_* or *NA_i_* = *NA_l_*, where the phase for the light-sheet pupil *p*_0_ is equal to zero and the phase for the detection PSF pupil *p_Z_* is an approximation of the optical aberrations written as an expansion in a Zernike polynomial basis. The Gaussian blur *g_σ_* in (2.2) is a technical detail that enables better fitting of the detection PSF *h* to the optical aberrations seen in bead data, an idea introduced in [26]. More details about the pupil functions and the aberration fitting using Zernike polynomials and the Gaussian blur *g_σ_* will be given in Sect. 2.3. In general, the NA of the excitation sheet is much lower than the NA of the detection lens. We note that the overall process is not translation invariant and cannot be modelled by a convolution operator.

Note that both the detection PSF *h* and the light-sheet PSF have a similar formulation derived from:

(2.5) $$\text{PSF}(x,y,z)=|\iint p(\kappa_{x},\kappa_{y})e^{2i\pi z\sqrt{{\left(n/\lambda_{i}\right)}^{2}-\kappa_{x}^{2}-\kappa_{y}^{2}}}e^{2i\pi (\kappa_{x}x+\kappa_{y}y)}\text{d}\kappa_{x}\text{d}\kappa_{y}{|}^{2},$$

which includes the pupil function for modelling aberrations and a defocus term before taking the Fourier transform (see, for example [27,28]). In addition, the actual light-sheet which illuminates a slice of the sample is obtained by rapid scanning of the illumination beam, which we model by *y*-averaging the illumination PSF *l_beam_* given in (2.3) and repeating it in the *y* direction for the full length of the sample.

In practice, the image formation process modelled by (2.1) is discretised at the point of recording by the camera sensor in the *xy* plane and by the step size of the light-sheet in the *z* direction. If the camera has a resolution of *N_x_* × *N_y_* pixels and the light-sheet illuminates the sample at *N_z_* distinct steps, the model (2.1) becomes:

(2.6) $${\tilde{f}}_{i,j,k}=\frac{1}{\tilde{C}}\sum_{i^{\prime }=1}^{N_{x}}\sum_{j^{\prime }=1}^{N_{y}}\sum_{k^{\prime }=1}^{N_{z}}{\tilde{l}}_{i^{\prime },j^{\prime },k^{\prime }}{\tilde{u}}_{i^{\prime },j^{\prime },k^{\prime }-k}{\tilde{h}}_{i-i^{\prime },j-j^{\prime },k^{\prime }},$$

for all *i* = 1*, … , N_x_, j* = 1*, … , N_y_, k* = 1*, … , N_z_*, and a normalisation constant $\tilde{C}$, where $\tilde{u},\tilde{f},\tilde{l},\tilde{h}\in {\mathbb{R}}^{N_{x}\times N_{y}\times N_{z}}$ are the discretised versions of *u, f , l, h*, respectively. Similarly, the sampling performed by the camera sensor leads to a discretisation of the Fourier space and the use of the discrete Fourier transform in the PSF and light-sheet models (2.2) and (2.3). Lastly, in our implementation we normalise $\tilde{h}$ so that $\sum_{i=1}^{N_{x}}\sum_{j=1}^{N_{y}}\sum_{k=1}^{N_{z}}{\tilde{h}}_{i,j,k}=1$ and choose the normalisation constant $\tilde{C}$ so that the norm of the resulting operator is equal to one.

### 2.2 Derivation of the Model

Let *l, u, h* be defined as in Sect. 2.1, with *h* and *l* centred at the origin and *l* translation invariant in the *y* direction and symmetric around the *yz* plane. For a fixed $z_{0}\in \left[-\frac{\Omega_{z}}{2},\frac{\Omega_{z}}{2}\right]$, we take the following steps, which replicate the inner workings of a light-sheet microscope:

1. Image the sample at *z* = *z*_0_: centre the sample *u* at *z*_0_ and multiply the result with the light-sheet *l*:
  (2.7) $$F(x,y,z;z_{0})=u(x,y,z-z_{0})\cdot l(x,y,z),$$
2. Convolve with the objective PSF *h*:
  (2.8) $$\begin{array}{l} C\left(x,y,z;z_{0}\right)\\ \;=F\left(x,y,z;z_{0}\right)*h\left(x,y,z\right)\\ \;=\int \int \int F\left(s,t,w;z_{0}\right)h\left(x-s,y-t,z-w\right)\text{d}s\text{d}t\text{d}w, \end{array}$$
3. Slice at *z* = 0:
  (2.9) $$f(x,y,z_{0})=[C(x,y,z;z_{0}){]}_{z=0},$$
  which leads to:
  (2.10) $$\begin{array}{c} f(x,y,z_{0})=\int \int \int \;u(s,t,w-z_{0})l(s,t,w)h(x-s,y\\ -t,-w)\text{d}s\text{d}t\text{d}w. \end{array}$$

This is the same as model (2.1), where we substitute *z* for *z*_0_. Note that, if there are no aberrations in *h* or other sources of asymmetry in the *z* direction, we could simply write *h*(*x* − *s, y* − *t, w*) instead.

For a discretisation of the domain using a 3D grid with *N_x_* × *N_y_* pixels and *N_z_* light-sheet steps, the forward model can be computed by following the three steps above for each *k* = 1*, … , N_z_*, where we perform the convolutions using the fast Fourier transform (FFT), resulting in a number of $O\left(N_{x}N_{y}N_{z}^{2}\log\left(N_{x}N_{y}N_{z}\right)\right)$ operations.

Alternatively, we can rewrite the last integral above as:

(2.11) $$f(x,y,z_{0})=\int K(x,y,w)*h(x,y,-w)\text{d}w,$$

where

(2.12) $$K(x,y,w)=l(x,y,w)u(x,y,w-z_{0}),$$

and the convolution in (2.11) is a 2D convolution in (*x, y*):

(2.13) $$\begin{array}{l} K\left(x,y,w\right)*h\left(x,y,-w\right)\\ \;=\int \int K\left(s,t,w\right)h\left(x-s,y-t,-w\right)\text{d}s\text{d}t. \end{array}$$

In terms of numbers of FFTs performed on a discretised *N_x_* × *N_y_* × *N_z_* grid, this alternative formulation requires $𝒪\left(N_{x}N_{y}N_{z}^{2}\log\left(N_{x}N_{y}\right)\right)$ operations.

### 2.3 Point Spread Function Model

While both the light-sheet profile and the detection PSF are based on the same model of a defocused system (2.5) introduced in [27], note that our definition of *h* in (2.2) includes an additional convolution operation with a Gaussian *g_σ_* and a pupil function *p_Z_* with a nonzero phase. Let us turn to why this is the case.

**Fig. 4:**
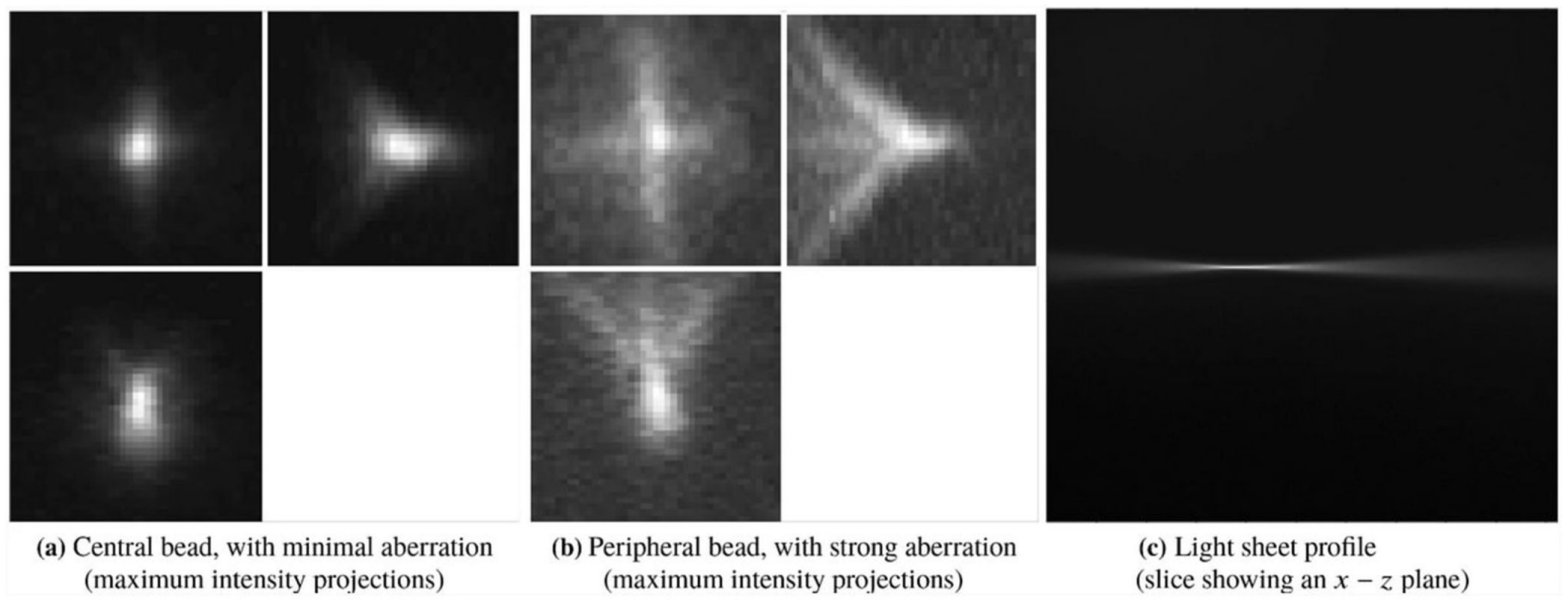
Examples of beads and light-sheet profile. The bead in **a** is cropped from the centre of Fig. 2a and the bead in **b** is cropped from the right-hand side of Fig. 2a. The maximum intensity projections are taken in the *x* − *y* plane (top left), the *z* − *y* plane (top right) and the *x* − *z* plane (bottom left)

It is well known that optical aberrations hamper results based on deconvolution with theoretical PSFs. In light-sheet microscopy, the effect of aberrations is more visible away from the centre, as shown for example in the bead image in Fig. 2, or in the more detailed example beads in Fig. 4. It is, therefore, required that we model the (spatially invariant) aberrations of the detection lens.

**Fig. 5:**
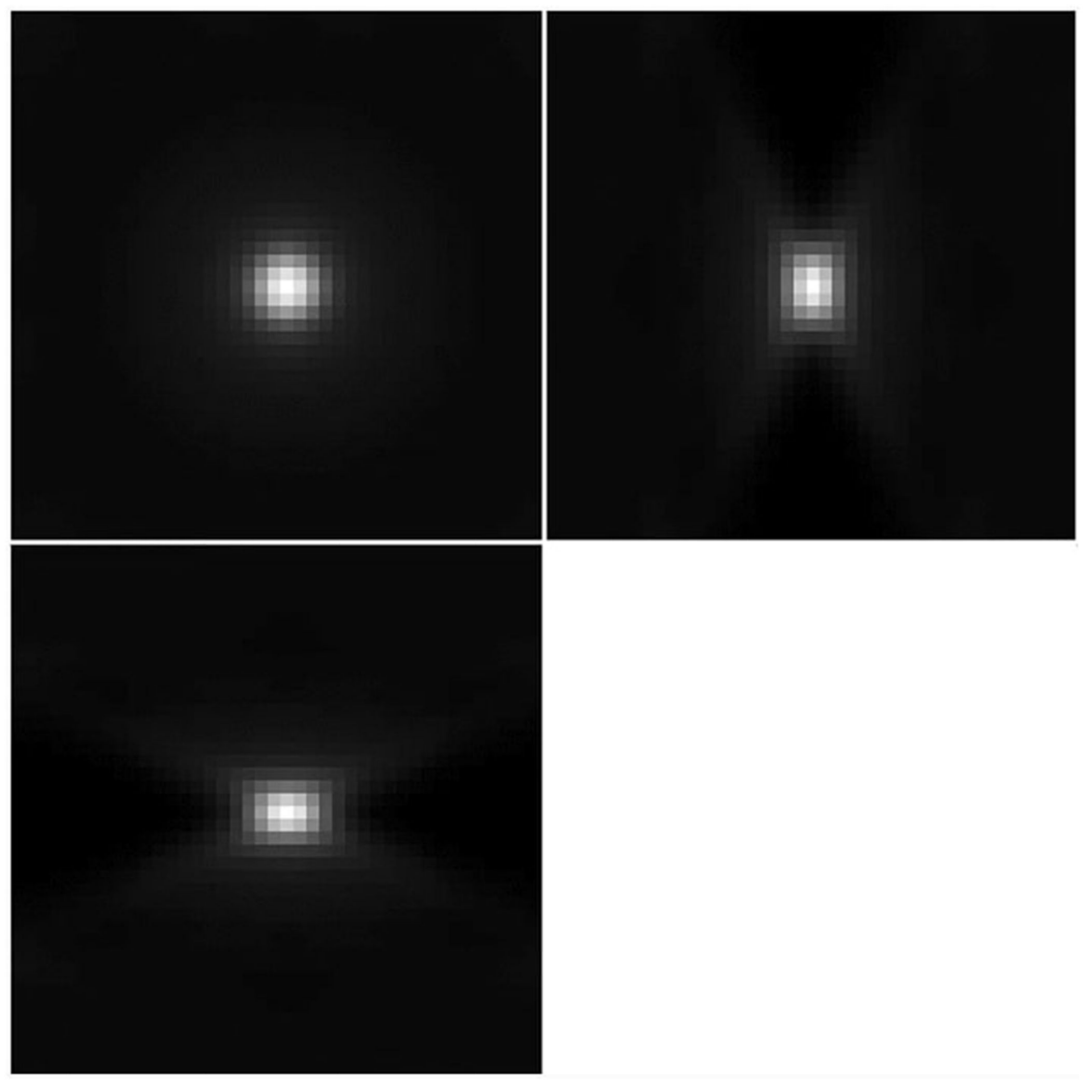
Objective PSF used in our model, with no aberrations (maximum intensity projections taken in the same way as in Fig. 4)

The general PSF model (2.5), with the phase of the pupil function equal to zero, does not take optical aberrations into account and therefore it is not an accurate representation of the objective PSF *h*. For example, a PSF calculated using (2.5) with zero phase of the pupil and the parameters of the detection objective, shown in Fig. 5, does not resemble the actual bead images in the data in Fig. 4.

There has been extensive work on the problem of phase reconstruction in the literature [26,29,30], but here we take a more straightforward approach using Zernike polynomials to include aberrations in the PSF [31], as follows. Let *h_z_* be the objective PSF calculated using (2.5) with Zernike polynomials in the phase of the pupil function:

(2.14) $$\begin{array}{c} h_{z}(x,y,z;c)\\ \;={\left|\iint p_{Z}(\kappa_{x},\kappa_{y};c)e^{2i\pi z\sqrt{{\left(n/\lambda_{h}\right)}^{2}-\kappa_{x}^{2}-\kappa_{y}^{2}}}e^{2i\pi (\kappa_{x}x+\kappa_{y}y)}\text{d}\kappa_{x}\text{d}\kappa_{y}\right|}^{2}, \end{array}$$

where *p_z_*(*κ_x_*, *κ_y_*; *c*) is the pupil function with *N_Z_* Zernike polynomials in the phase:

(2.15) $$\begin{array}{l} p_{z}\left(\kappa_{x},\kappa_{y};c\right)\\ \;=\{\begin{array}{ll} e^{2i\pi }\sum_{j=1}^{N_{Z}}c_{j}Z_{j}\left(\kappa_{x},\kappa_{y}\right) & \text{for}\;\sqrt{\kappa_{x}^{2}+\kappa_{y}^{2}}\leq NA/\lambda_{h},\\ 0, & \text{otherwise}, \end{array} \end{array}$$

and *c* = [*c*_1_*, … , c_Nz_*]^*T*^ are coefficients corresponding to the polynomials for some integer *N_Z_ >* 0.

Moreover, let *h_zb_* be the blurred PSF obtained by convolving *h_z_* with a Gaussian *g_σ_* with width *σ*:

(2.16) $$h_{zb}(x,y,z;c,\sigma )=h_{z}(x,y,z;c)*g_{\sigma }.$$

This allows us to obtain a better approximation of the objective PSF [26]. The parameters *c* and *σ* are calculated by solving the least-squares problem

(2.17) $$\underset{c,\sigma }{\text{min}}\;\|h_{zb}(c,\sigma )*b-h_{\mathrm{bead}}{\|}_{2}^{2}$$

(2.18) $$\text{subject}\;\text{to}\;c\in [-B_{Z},B_{Z}{]}^{N_{Z}},\sigma >0,$$

for some *B_Z_ >* 0, where *h_bead_* is a bead image containing the aberrations that one wants to capture in the fitted detection PSF (for example the bead in Fig. 4b) and *b* is equal to one inside the sphere of the radius equal to the radius of the bead (a parameter that is provided) and zero outside the sphere. This takes into account the non-negligible size of the beads used to generate the data.

In the implementation of the fitting procedure, we normalise both the bead image *h_bead_* and the simulated PSF *h_zb_* by their maximum values before calculating their error, and we include two additional parameters, scaling and shift, to ensure a better fit of the intensity values (not shown here for simplicity of the presentation).

**Fig. 6:**
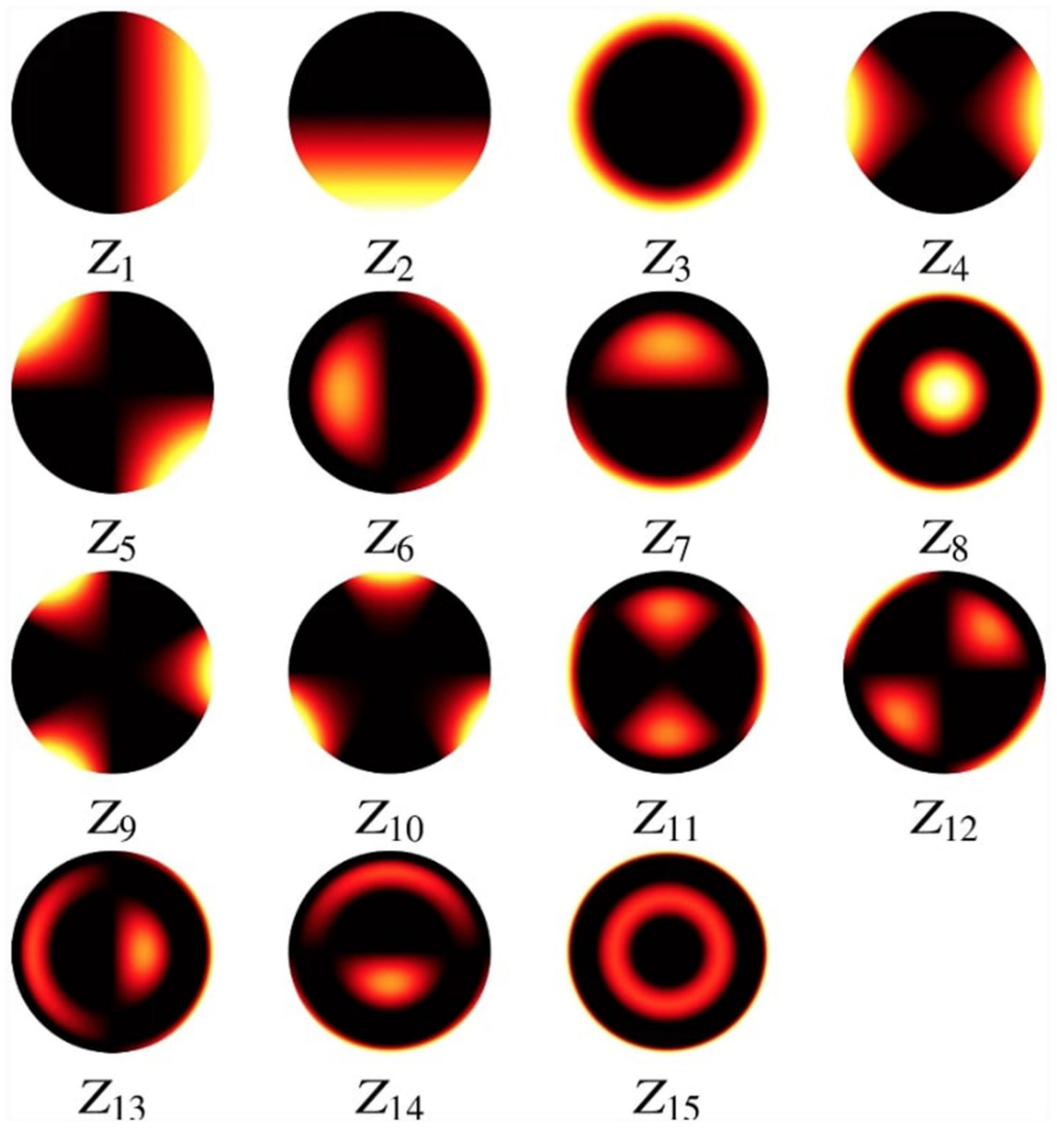
The Zernike polynomials used in the PSF *h_z_*, with image range [−1, 1]

**Fig. 7:**
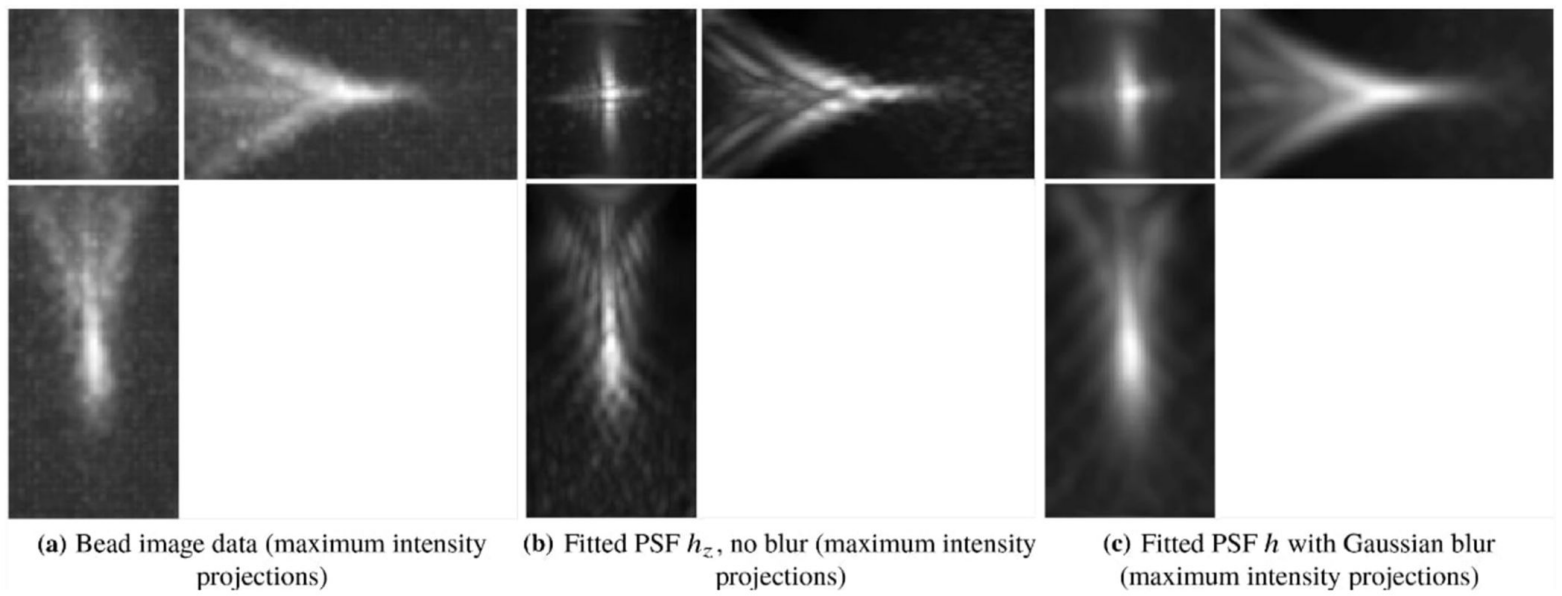
Fitted PSF using Zernike polynomials. In panel **c**, we can see the benefits of using the Gaussian blur *g_σ_* in obtaining an accurate approximation of the bead in **a**

The best choice of the number of Zernike polynomial basis elements *N_Z_* and the boundary *B_Z_* of the coefficients *c* depend on the data *h_bead_* and how well the fit is required to be in the deconvolution step. In general, at least the first 15 Zernike polynomials are needed to capture the main optical aberrations such as spherical and astigmatism. In our experiments, for the bead shown in Fig. 4b, we found that *N_Z_* = 15 and *B* = 3 are an appropriate choice. The Zernike polynomials used and their resulting corresponding coefficients are shown in Table 1 and in Fig. 6. The resulting PSF is the detection PSF model (2.2) and is shown in Fig. 7.

Finally, we note that a more thorough approach to choosing the Zernike basis is possible, by using multiple bead images for fitting the Zernike coefficients and comparing the quality of the fit for different values of *N_Z_* and *B_Z_*. Alternatively, one could average multiple beads and perform the fitting procedure described above on the averaged bead. In both cases, it is worth mentioning that, since the optical aberrations vary within the sample image, we would only be able to fit the general shape of the PSF rather than the sharper features present in each bead, effectively fitting the low frequency information in the beads. In the end, this would achieve a similar effect to the Gaussian blur *g_σ_* that we use in the fitting process. Moreover, one can employ more advanced techniques such as the ones described in [26,29,30] for estimating the pupil function, which can be plugged in to our image formation model. However, such an analysis focused on the pupil function is beyond the scope of the present work.

### 2.4 Convolution with Spatially Varying Kernel

Having introduced the image formation model for a light-sheet microscope (2.1) as well as the models for the individual PSFs, it is worth expanding on the source of spatial variability that we tackle in this work.

First, note that with a change of variable *w* → *w* + *z*, we can rewrite the model (2.1) as:

$$\begin{array}{c} f(x,y,z)=\int \int \int \;u(s,t,w)l(s,t,w+z)h(x-s,y-t,\\ -w-z)\text{d}s\text{d}t\text{d}w, \end{array}$$

so f (*x, y, z*) is the convolution of *u*(*x, y, z*) with the spatially varying kernel $\tilde{h}$(*x, y, z*; *s, t, w*):

(2.19) $$f(x,y,z)=\int \int \int \;u(s,t,w)\tilde{h}(x,y,z;s,t,w)\text{d}s\text{d}t\text{d}w,$$

where

(2.20) $$\tilde{h}(x,y,z;s,t,w)=l(s,t,w+z)h(x-s,y-t,-w-z)$$

gives the expression of a kernel $\tilde{h}(x,y,z;\cdot ,\cdot ,\cdot )$ which varies with its centre (*x, y, z*).

Therefore, the model presented in this section describes the spatial variation of the *overall* PSF of the system $\tilde{h}$, as a consequence of the interaction between the light-sheet beam PSF *l* and the detection PSF *h*. We highlight that *l* and *h* are not themselves spatially varying. By the process described in Sect. 2.2, this interaction (and spatial variability) is modelled explicitly. This is in contrast to approaches such as [16], where the spatial variability of the PSF is learned from the data and encoded in the black-box mechanism of an artificial neural network.

In practice, a second source of spatial variability of the PSF may be the detection PSF *h*, due to the optical aberrations that can vary within the sample image. As described in Sect. 2.3, in this work we do not account for this potential spatial variability of *h*, and we fit one pupil function to the bead data.

## 3 Inverse Problem

### 3.1 Problem Statement

In this section we formally state the inverse problem of deblurring a light-sheet microscopy image. Let $\Omega \subset {\mathbb{R}}^{3}$ be a bounded Lipschitz domain and let *L* : L^*p*^(Ω) → L^2^(Ω) be the forward operator defined by (2.1). Here 1 < *p* < 3/2 is chosen such that the embedding of the BV space is compact [32]. Clearly, *L* is linear.

We consider the following inverse problem

(3.1) $$Lu=\bar{f},$$

where $\bar{f}\in {\text{L}}^{2}(\Omega )$ is the exact (noise-free) data. As outlined in Sect. 2.1, the measurements in light microscopy are corrupted by a combination of Poisson and Gaussian noise. More precisely, the measurement is given by *f* = *v* + *w*, where $v\;\sim \;Pois\;(\bar{f})$ is a Poisson distributed random variable with mean $\bar{f}$ and *w* represents additive zero-mean Gaussian noise. We do not model Gaussian noise statistically and instead, in the spirit of (deterministic) variational regularisation, assume that *w* ∈ L^2^(Ω) is a fixed perturbation with $\|w{\|}_{{\text{L}}^{2}(\Omega )}\leq \sigma_{G}$ for some known *σ_G_ >* 0. Poisson noise is typically modelled using the Kullback–Leibler divergence as the data fidelity term [33,34].

Let us give a brief justification of the inverse problem formulation described in this section [9,35], from a Bayesian perspective. First, by using the Poisson and Gaussian probability density functions, we have that $p(v|u)=\frac{{\left(Lu\right)}^{v}e^{-(Lu)}}{v!}$, and $p(f|v)=\frac{1}{\sqrt{2\pi }\sigma_{G}}e^{-\frac{1}{2}{\left(\frac{f-v}{\sigma_{G}}\right)}^{2}}$, and from Bayes’ theorem and conditional probability:

(3.2) $$p(u,v|f)=\frac{p(f|v)p(v|u)p(u)}{p(f)},$$

where we used that *p*(*f* | *u*, *v*) = *p*(*f* | *v*). Moreover, we assume that the prior is a Gibbs distribution *p*(*u*) = *e*^−*α𝒥* (*u*)^ for a convex functional *𝒥* (*u*), which we will introduce later. To obtain a maximum *a posteriori* estimation of *u* and *v* (i.e. maximise the posterior distribution *p*(*u*, *v*| *f*)), we take the minimum of the negative log of (3.2) and, after discarding the denominator *p*(*f*) and using the Stirling approximation for the factorial log *v*! = *v* log *v* − *v*, we obtain the minimisation problem:

(3.3) $$\text{arg}\;{\text{min}}_{u,v}\alpha 𝒥(u)+\frac{1}{2\sigma_{G}^{2}}∥f-v{∥}^{2}+v\;\text{log}\frac{v}{Lu}+Lu-v,$$

where the first term is the regularisation term and the remaining terms form the data fidelity term.

We will now describe the formal mathematical setting for (3.3) in the context of variational regularisation. This will allow us to show well-posedness of the model, establish convergence rates of the solution with respect to the noise in the measurements and to introduce a discrepancy principle for choosing the value of the regularisation parameter *α*.

First, note that in (3.3), we can perform the minimisation over *v* only on the data fidelity part of the objective, which can be written as an infimal convolution of the two separate Gaussian and Poisson fidelities. The infimal convolution of two functionals *φ*_1_, *φ*_2_ on L^2^ is defined as^1^:

(3.4) $$(\varphi_{1}□\varphi_{2})(f)=\underset{v\in {\text{L}}^{2}(\Omega )}{\text{inf}}\{\varphi_{1}(f-v)+\varphi_{2}(v)\},$$

for *f* ∈ L^2^(Ω). Therefore, we define the following data fidelity term, as proposed in [9]:

(3.5) $$\text{Φ}(\bar{f},f)≔\underset{v\in {\text{L}}_{+}^{2}(\Omega )}{\text{inf}}\{\frac{1}{2}∥f-v{∥}_{{\text{L}}^{2}}^{2}+D_{KL}(v,\bar{f})\},$$

for *f* ∈ L^2^(Ω) and $\bar{f}\in {\text{L}}_{+}^{1}(\Omega )$, where ${\text{L}}_{+}^{1,2}(\Omega )$ denotes the positive cone in L^1,2^(Ω) (that is, functions *f* ∈ L^1,2^(Ω) such that *f* ≥ 0 a.e.) and *D_KL_* denotes the Kullback–Leibler divergence, which we define as follows

(3.6) $$\begin{array}{ll} D_{KL}(v,\bar{f})≔\{\begin{array}{l} \int_{\Omega }(\bar{f}(x)-v(x)+v(x)\log\frac{v(x)}{\bar{f}(x)})\text{d}x,\\ \;\text{if}\;v,\bar{f}\geq 0\wedge \int_{\Omega }v\text{d}x=\int_{\Omega }\bar{f}\text{d}x=1,\\ +\infty ,\;\text{otherwise}, \end{array} & \\ \;=\{\begin{array}{l} \int_{\Omega }v(x)(\text{log}\;v(x)-\text{log}\;\bar{f}(x))\text{d}x,\\ \;\text{if}\;v,\bar{f}\geq 0\wedge \int_{\Omega }v\text{d}x=\int_{\Omega }\bar{f}\text{d}x=1,\\ +\infty ,\;\text{otherwise}. \end{array} & \end{array}$$

We note that |*∫*_Ω_ *v*(*x*) log *v*(*x*) d*x*| < ∞ for *v* ∈ L^2^, since L^2^ is continuously embedded into the Orlicz space L log L of functions of finite entropy [36,37]

(3.7) $$\text{L}\log\text{L}(\Omega )≔\{f\in {\text{L}}^{1}(\Omega ):\int_{\Omega }|f(x)|{\left(\log|f(x)|\right)}_{+}\text{d}x<\infty \},$$

where (·)_+_ = max{·, 0} denotes the positive part.

A proof of the following result can be found in [9], but we provide it here for readers’ convenience.

**Proposition 3.1** (Exactness of the infimal convolution) *For any* $\bar{f}\in {\text{L}}_{+}^{1}$ *such that* $\int_{\Omega }\bar{f}\text{d}x=1$, there exists a unique solution $v^{*}=v^{*}(\bar{f})$ of (3.5)*, that is, the infimal convolution is exact. Moreover, the functional* $\Phi (\bar{f},\cdot ):{\text{L}}^{2}\to {\mathbb{R}}_{+}\cup$ {+∞} *is proper, convex and lower semicontinuous.*

***Proof*** Fix $\bar{f}\in {\text{L}}_{+}^{1}$ such that $\int_{\Omega }\bar{f}\text{d}x=1$. Then, (3.5) is the infimal convolution of the following two functionals on L^2^

$$\begin{array}{c} \varphi (g)≔\chi_{{\text{L}}_{+}^{2}}(g)+D_{KL}(g,\bar{f}),\\ \psi (g)≔\frac{1}{2}∥g{∥}_{{\text{L}}^{2}}^{2},\;g\in {\text{L}}^{2}(\Omega ), \end{array}$$

where *χ* denotes the characteristic function. The function *φ* is proper, convex, non-negative and lower semicontinuous, while *ψ* is proper, convex, lower semicontinuous and coercive. Therefore, by [38, Prop. 12.14], the infimal convolution is exact and is itself a proper, convex and lower semicontinuous function. Uniqueness follows from strict convexity of *ψ*.

Now we turn our attention to the lower semicontinuity of the functional Φ(·, *f*) in its first argument.

**Proposition 3.2** (Lower semicontinuity) *For any* $f\in L_{+}^{2}(\Omega )$ *such that* *∫*_Ω_ *f* d*x* = 1 *the functional* Φ(·, *f*): *L*^1^(Ω) → ℝ_+_ ∪{+∞} *is lower semicontinuous.*

***Proof*** We have

$$\begin{array}{l} \Phi \left(g,f\right)=\underset{v\in {\text{L}}_{+}^{2}\left(\Omega \right)}{\text{inf}}\left\{\frac{1}{2}{\left\|f-v\right\|}_{{\text{L}}^{\text{2}}}^{2}+D_{KL}\left(v,g\right)\right\}\\ \;=\frac{1}{2}{\left\|f-v*\left(g\right)\right\|}_{{\text{L}}^{\text{2}}}^{2}+D_{KL}\left(v*\left(g\right),g\right)\\ \;=\frac{1}{2}{\left\|f-v*\left(g\right)\right\|}_{{\text{L}}^{\text{2}}}^{2}+\\ \;+\int_{\Omega }v\left(x\right)\left(\text{log}\;v\left(x\right)-\text{log}\;\bar{f}\left(x\right)\right)\text{d}x+\chi 𝒞\left(g\right), \end{array}$$

where *g* ∈ L^1^(Ω), v^*^(*g*) is as defined in Proposition 3.1 and $𝒞≔\{g\in L_{+}^{1}\left(\Omega \right):\int_{\Omega }g\text{d}x=1\}.$ The characteristic function is lower semicontinuous because *𝒞* is closed in L^1^ and the rest is lower semicontinuous by [9, Thm. 4.1].

The following fact is easily established.

**Proposition 3.3** *The operator L* : L^*p*^(Ω) → L^1^(Ω) *defined in* (2.1) *is continuous for any p* ≥ 1*. Moreover, if l and h are non-negative and have overlapping support:*

supp(l)∩supp(h)≠∅,

*then* **1** ∉ *𝒩* (*L*), *where* **1** *is the constant one function and 𝒩*(*L*) *is the null space of L*.

***Proof*** By (2.1), we have

$$\begin{array}{c} Lu(x,y,z)\\ \;=\int_{\Omega }l(s,t,w)h(x-s,y-t,w)u(s,t,w-z)\text{d}\mu_{stw}, \end{array}$$

where d*μ_stw_* ≔ d*s*d*t*d*w*. Noting that the light-sheet PSF *l* and detection PSF *h* are bounded from above by some *C*_1_*, C*_2_ > 0, we have that:

$$\begin{array}{l} {\left\|Lu\right\|}_{{\text{L}}^{1}}\\ \;=\int_{\Omega }|\int_{\Omega }l\left(s,t,w\right)h\left(x-s,y-t,w\right)u\left(s,t,w-z\right)d\mu_{stw}|d\mu_{xyz}\\ \;=\int_{\Omega }|\int_{\Omega }l\left(s,t,w^{\prime }+z\right)h\\ \;\left(x-s,y-t,w^{\prime }+z\right)u\left(s,t,w^{\prime }\right)d\mu_{stw^{\prime }}{|d\mu }_{xyz}\\ \;(\text{by}\;w^{\prime }=w-z)\\ \;\leq C_{1}C_{2}\int_{\Omega }|\int_{\Omega }u\left(s,t,w^{\prime }\right)d\mu_{stw^{\prime }}|d\mu_{xyz}\\ \;=C_{1}C_{2}\left|\Omega \right||\int_{\Omega }u\left(s,t,w^{\prime }\right)d\mu_{stw^{\prime }}|\\ \;\leq C\left(p\right){\left\|u\right\|}_{L^{p}}, \end{array}$$

where in the last inequality we applied Hölder’s inequality and *C*(*p*) is a constant that depends on *p* (as well as *C*_1,2_ and Ω). Hence, we obtain the first claim.

For the second claim, we observe that

$$L1(x,y,z)=\int_{\Omega }l(s,t,w)h(x-s,y-t,w)\text{d}\mu_{stw}\geq 0.$$

Consider

$$\begin{array}{l} \int_{\Omega }L1(x,y,z)\text{d}\mu_{xyz}\\ \;=\int_{\Omega }\int_{\Omega }l(s,t,w)h(x-s,y-t,w)\text{d}\mu_{stw}\text{d}\mu_{xyz} \end{array}$$

and let *B_l,h_* ⊂ supp *l* ⋂ supp *h*. Then, since both *l* and *h* are non-negative on Ω, from the last equality above we have that:

$$\int_{\Omega }L1(x,y,z)\text{d}\mu_{xyz}\geq \int_{B_{l,h}}l(x,y,z)h(x,y,z)\text{d}\mu_{xyz}>0,$$

which proves the second claim.

***Remark 3.1*** Our setting with the measured data *f* ∈ L^2^(Ω) differs slightly from [9], where *f* ∈ L^∞^(Ω) was assumed.

We will consider the following variational regularisation problem

(3.8) $$\underset{u\in {\text{L}}_{+}^{p}(\Omega )}{\text{min}}\Phi (f,Lu)+\alpha J(u),$$

where Φ is the infimal convolution fidelity as defined in (3.5), $J:{\text{L}}^{p}\to {\mathbb{R}}_{+}\cup \{+\infty \}$ is a regularisation functional, $\alpha \in {\mathbb{R}}_{+}$ is a regularisation parameter and 1 < *p* < 3/2. Without loss of generality, we assume that $\int_{\Omega }\bar{f}\text{d}x=1$.

As the regulariser 𝒥, we choose the total variation [39]

$$J(u)=\text{TV}(u)≔\underset{\begin{array}{c} \xi \in C_{0}^{\infty }(\Omega ,{\mathbb{R}}^{3})\\ ∥\xi {∥}_{\infty }\leq 1 \end{array}}{\text{sup}}\int_{\Omega }u\;\text{div}(\xi )\text{d}x.$$

By the Rellich–Kondrachov theorem, the space

$$\begin{array}{l} \text{BV}(\Omega )≔\{u\in {\text{L}}^{1}(\Omega ):\text{TV}(u)<\infty \},\\ \;∥u{∥}_{\text{BV}}≔∥u{∥}_{{\text{L}}^{1}}+\text{TV}(u), \end{array}$$

is compactly embedded into L^*p*^(Ω) for 1 ≤ *p <* 3/2 and continuously embedded into L^3/2^(Ω) since Ω ⊂ ℝ^3^. Therefore, we consider TV: ${\text{L}}^{p}\to {\mathbb{R}}_{+}\cup \{+\infty \}$

$$\text{TV}(u)≔\{\begin{array}{ll} \underset{\begin{array}{c} \xi \in C_{0}^{\infty }(\Omega ,{\mathbb{R}}^{3})\\ ∥\xi {∥}_{\infty }\leq 1 \end{array}}{\text{sup}}\int_{\Omega }u\;\text{div}(\xi )\text{d}x, & u\in \text{BV}(\Omega ),\\ \infty , & u\in {\text{L}}^{p}(\Omega )\backslash \text{BV}(\Omega ). \end{array}$$

We will denote by $u_{\text{TV}}^{†}$ the TV-minimising solution of (3.1), i.e. a solution that satisfies

$$Lu_{\text{TV}}^{†}=\bar{f}\;\text{and}\;\text{TV}(u_{\text{TV}}^{†})\leq \text{TV}(u)\;\text{for}\;\text{all}\;u\;\text{s}.\text{t}\;.Lu=\bar{f}.$$

The existence of such solution is obtained by standard arguments [40]. We will make the reasonable assumption that the TV-minimising solution is positive, i.e. $u_{\text{TV}}^{†}\geq 0$ a.e. Due to the positivity of the kernels involved in (2.1), it is clear that $u_{\text{TV}}^{†}\geq 0$ implies $Lu_{\text{TV}}^{†}=\bar{f}\geq 0$.

Since by Proposition 3.1 the infimal convolution (3.5) is exact, we can equivalently rewrite (3.8) as follows

(3.9) $$\underset{\begin{array}{c} u\in {\text{L}}_{+}^{p}(\Omega )\\ \nu \in {\text{L}}_{+}^{2}(\Omega ) \end{array}}{\text{min}}\frac{1}{2}∥f-\nu {∥}_{{\text{L}}^{2}}^{2}+D_{KL}(\nu ,Lu)+\alpha J(u).$$

Existence of minimisers in (3.8) and (3.9) is obtained by standard arguments [9, Thm. 4.1].

**Proposition 3.4** *Each of the optimisation problems* (3.8) *and* (3.9) *admits a unique minimiser*.

We will also need the following coercivity result.

**Proposition 3.5** *The functional* $\Phi (f,\cdot ):{\text{L}}^{1}(\Omega )\to {\mathbb{R}}_{+}\cup$ {+∞} *is strongly coercive with exponent* 2*, i.e. there exists a constant C* > 0 *such that*

$$\Phi (f,g)\geq C∥g-f{∥}_{{\text{L}}^{1}}^{2},\;g\in {\text{L}}^{1}(\Omega ).$$

***Proof*** Using Pinsker’s inequality for the Kullback–Leibler divergence, we get

$$\begin{array}{l} \Phi (f,g)\geq \underset{\nu \in {\text{L}}_{+}^{2}}{\text{inf}}\frac{1}{2}∥\nu -f{∥}_{{\text{L}}^{2}}^{2}+D_{KL}(\nu ,g)\\ \;\geq \underset{\nu \in {\text{L}}_{+}^{2}}{\text{inf}}\frac{1}{2}∥\nu -f{∥}_{{\text{L}}^{2}}^{2}+∥g-\nu {∥}_{{\text{L}}^{1}}^{2}\\ \;\geq 2C\underset{\nu \in {\text{L}}_{+}^{2}}{\text{inf}}∥\nu -f{∥}_{{\text{L}}^{1}}^{2}+∥g-\nu {∥}_{{\text{L}}^{1}}^{2} \end{array}$$

for some *C* > 0. Note that Pinsker’s inequality assumes that *f*, *g* ≥ 0 and inf_Ω_ *f* d*x* = *∫*_Ω_ *g*d*x* = 1, which we ensure by definition in (3.6).

Now, using the inequality $\frac{1}{2}(a+b{)}^{2}\leq a^{2}+b^{2}$ that holds for all a,b∈ℝ and the triangle inequality, we obtain the claim

$$\begin{array}{l} \Phi (f,g)\geq C\underset{\nu \in {\text{L}}_{+}^{2}}{\text{inf}}{\left(∥\nu -f{∥}_{{\text{L}}^{1}}+∥g-\nu {∥}_{{\text{L}}^{1}}\right)}^{2}\\ \;\geq C\underset{\nu \in {\text{L}}_{+}^{2}}{\text{inf}}∥\nu -f+g-\nu {∥}_{{\text{L}}^{1}}^{2}\\ \;=C∥g-f{∥}_{{\text{L}}^{1}}^{2}. \end{array}$$

### 3.2 Convergence Rates

Our aim in this section is to establish convergence rates of minimisers of (3.8) as the amount of noise in the data decreases. But first we need to specify what we mean by the amount of noise in our setting.

We argue as follows. Since the noise in the measurement is generated sequentially, i.e. photo-electrons are first counted by the sensor leading to a Poisson noise and later they are collected by the electronic circuit generating an additive Gaussian noise, for any exact data $\bar{f}$ there exists $\bar{z}∼Pois(\bar{f})$ such that $D_{KL}(\bar{z},\bar{f})\leq \gamma$, where *γ >* 0 depends on the exposure time *t* and vanishes as *t* → ∞ [33]. Further, there exists *w* ∈ L^2^(Ω) with $\|w{\|}_{{\text{L}}^{2}}\leq \sigma_{G}$ such that $f=\bar{z}+w$. Since $\bar{z}\geq 0$ is feasible in (3.5), we get the following upper bound on the fidelity term (3.5) evaluated at the measurement *f* and the exact data $\bar{f}$

(3.10) $$\begin{array}{l} \Phi (\bar{f},f)\leq \frac{1}{2}∥f-\bar{z}{∥}_{{\text{L}}^{2}}^{2}+D_{KL}(\bar{z},\bar{f})\\ \;=\frac{1}{2}∥w{∥}_{{\text{L}}^{2}}^{2}+D_{KL}(\bar{z},\bar{f})\\ \;\leq \frac{\sigma_{G}^{2}}{2}+\gamma . \end{array}$$

The standard tool for establishing convergence rates are Bregman distances associated with the regulariser 𝒥. We briefly recall the necessary definitions.

**Definition 3.1** Let *X* be a Banach space and $J:X\to {\mathbb{R}}_{+}\cup$ {+∞} a proper convex functional. The generalised Bregman distance between *x, y* ∈ *X* corresponding to the subgradient *q* ∈ ∂𝒥 (*y*) is defined as follows

$$D_{J}^{q}(x,y)≔J(x)-J(y)-\langle q,x-y\rangle .$$

Here ∂𝒥 (*ν*) denotes the subdifferential of 𝒥 at *y* ∈ *X*. If, in addition, *p* ∈ ∂𝒥 (*x*), the symmetric Bregman distance between *x, y* ∈ *X* corresponding to the subgradients *p, q* is defined as follows

$$D_{J}^{p,q}(x,y)≔D_{J}^{q}(x,y)+D_{J}^{p}(y,x)=\langle p-q,x-y\rangle .$$

To obtain convergence rates, an additional assumption on the regularity of the TV-minimising solution, called the *source condition*, needs to be made. We use the following variant [41].

**Assumption 3.1** (Source condition) There exists an element *μ*^†^ ∈ *L*^∞^(Ω) such that

$$q^{†}≔L^{*}\mu^{†}\in \partial J(u_{\text{TV}}^{†}).$$

#### 3.2.1 Parameter Choice Rules

Let us summarise what we know about the fidelity function Φ as defined in (3.5), the regularisation functional TV and the forward operator *L*:

– Φ(*f*, ·*)* is proper, convex and coercive (Proposition 3.5) in L^1^(Ω);
– Φ(·, ·*)* is jointly convex [42] and lower semicontinuous (Propositions 3.1 and 3.2);
– Φ(*f*, g) = 0 if and only if *f* = *g*;
– TV: ${\text{L}}^{1}(\Omega )\to \mathbb{R}\cup \{+\infty \}$ is proper, convex and lower semicontinuous [32] and its null space is given by 𝒩(TV) = span{**1**}, where **1** denotes the constant one function;
– TV is coercive on the complement of its null space in L^1^(Ω) [32];
– *L* : L^*p*^(Ω) → L^1^(Ω) is continuous and 𝒩(TV) ⋂ 𝒩(L) = {0} (Proposition 3.3).

Using these facts and slightly modifying the proofs from [43], we obtain the following

**Theorem 3.2** (Convergence rates under a priori parameter choice rules) *Let assumptions made in* Sect. 3.1 *hold and let the source condition* (Theorem 3.1) *be satisfied at the* TV-*minimising solution $u_{\text{TV}}^{†}$. Let u_σG,γ_ be a solution of* (3.8) *and let α be chosen such that*

$$\alpha (\sigma_{G},\gamma )=O(\sigma_{G}+\sqrt{\gamma }).$$.

*Then,*

$$D_{\text{TV}}^{q^{†}}(u_{\sigma_{G},\gamma },u_{\text{TV}}^{†})=O(\sigma_{G}+\sqrt{\gamma }),$$

*where q*^†^ = L^*^μ^†^ *is the subgradient from* Theorem 3.1 *and σ_G_, γ >* 0 *are as defined in* (3.10).

***Proof*** The proof is similar to [43, Thm. 3.9].

In a similar manner, we can obtain convergence rates for an a posteriori parameter choice rule known as the discrepancy principle [44–46]. Let *f* be the noisy data and *δ >* 0 the amount of noise such that $\Phi (\bar{f},f)\leq \delta$, where Φ is as defined in (3.5). In our case, $\delta =\frac{\sigma_{G}^{2}}{2}+\gamma$ by (3.10). The discrepancy principle amounts to selecting *α* = *α*(*f*, *δ*) such that

(3.11) $$pg\alpha =\text{sup}\{\alpha >0:\Phi (Lu^{\alpha },f)\leq \tau \delta \},$$

where *u^α^* is the regularised solution corresponding to the regularisation parameter *α* and *τ >* 1 is a parameter.

Again, slightly modifying the proofs from [43], we obtain the following

**Theorem 3.3** (Convergence rates under the discrepancy principle) *Let assumptions made in* Sect. 3.1 *hold and let the source condition* (Theorem 3.1) *be satisfied at the* TV-*minimising solution* $u_{\text{TV}}^{†}$*. Let u_σG,γ_ be a solution of* (3.8) *with α chosen according to the discrepancy principle* (3.11). *Then,*

$$D_{\text{TV}}^{q^{†}}(u_{\sigma_{G},\gamma },u_{\text{TV}}^{†})=O(\sigma_{G}+\sqrt{\gamma }),$$

*where q*^†^ = L*μ^†^ *is the subgradient from* Theorem 3.1 *and σ_G_, γ >* 0 *are as defined in* (3.10).

***Proof*** The proof is similar to [43, Thm. 4.10].

## 4 Solving the Minimisation Problem

### 4.1 PDHG for Infimal Convolution Model

In practice, due to the joint convexity of the Kullback–Leibler divergence, we solve the minimisation problem (3.9), where we treat the reconstructed sample *u* and the Gaussian denoised image *v* jointly and, in addition, we impose lower and upper bound constraints on *u* and *v* by including the corresponding characteristic functions in the objective:

(4.1) $$\begin{array}{c} \underset{u,v}{\min}\alpha \text{TV}(u)+\frac{1}{2\sigma_{G}^{2}}∥f-v{∥}_{2}^{2}+D_{KL}(v,Lu)+\chi_{{\left[l_{1},l_{2}\right]}^{2N}}({\left[u,v\right]}^{T}). \end{array}$$

Note that the objective function in (4.1) is a sum of convex functions (the Kullback–Leibler divergence *D_KL_* is jointly convex [47]), and therefore is itself convex. We then write the problem (4.1) as:

(4.2) $$\underset{w}{\min}G(w)+\sum_{i=1}^{m}H_{i}(L_{i}w),$$

where we solve for $w=\left[\begin{array}{l} u\\ v \end{array}\right]$, *m* = 3 and:

(4.3) $$G(w)=\chi_{{\left[l_{1},l_{2}\right]}^{2N}}\left(\left[\begin{array}{c} u\\ v \end{array}\right]\right),$$

(4.4) $$\begin{array}{cc} H_{1}(\cdot )=\frac{1}{2\sigma_{G}^{2}}{\left\|\cdot -f\right\|}_{2}, & L_{1}=\left[\begin{array}{cc} 0 & 1 \end{array}\right], \end{array}$$

(4.5) $$\begin{array}{cc} H_{2}(w)=D_{KL}\left(v,u\right), & L_{2}=\left[\begin{array}{cc} L & 0\\ 0 & 1 \end{array}\right], \end{array}$$

(4.6) $$\begin{array}{cc} H_{3}(\cdot )=\alpha {\left\|\cdot \right\|}_{1}, & L_{3}=\left[\begin{array}{cc} \nabla_{x} & 0\\ \nabla_{y} & 0\\ \nabla_{z} & 0 \end{array}\right], \end{array}$$

where *L* is the forward operator corresponding to the image formation model from Sect. 2.1.

Rather than solving the problem (4.2) directly, a common approach is to reformulate it as a saddle point problem using the Fenchel conjugate G^*^(*y*) = sup_*z*_ 〈*z, y*〉 − *G(z)*. For proper, convex and lower semicontinuous function *G*, we have that G^**^ = *G*, so (4.2) can be written as the saddle point problem

(4.7) $$\underset{w}{\min}\underset{y_{1},\ldots ,y_{m}}{\sup}G(w)+\sum_{i=1}^{m}\langle y_{i},L_{i}x\rangle -H_{i}^{*}(y_{i}),$$

and by swapping the min and the sup and applying the definition of the convex conjugate G^*^, one obtains the dual of (4.2):

(4.8) $$\underset{y_{1},\ldots ,y_{m}}{\max}-G^{*}(-\sum_{i=1}^{m}L_{i}^{*}y_{i})-\sum_{i=1}^{m}H_{i}^{*}(y_{i}).$$

The saddle point problem (4.7) is commonly solved using the primal–dual hybrid gradient (PDHG) algorithm [6,10,11], and by doing so, both the primal problem (4.2) and the dual (4.8) are solved. We apply the variant of PDHG from [48], which accounts for the sum of composite terms *H_i_* ◦ *L_i_*. Given an initial guess for *(w*_0_*, y*_1,0_*, … , y_m,_*_0_*)* and the parameters *σ, τ >* 0, and *ρ* ∈ [ϵ, 2 − ϵ] for some ∈ *ϵ* 0, each iteration *k* ≥ 0 consists of the following steps:

(4.9) $$\begin{array}{l} 1.{\tilde{w}}_{k+1}≔\text{pro}{\text{x}}_{\tau G}(w_{k}-\tau \sum_{i=1}^{m}L_{i}^{*}y_{i,k}),\\ 2.w_{k+1}≔\rho_{k}{\tilde{w}}_{k+1}+(1-\rho_{k})w_{k},\\ \begin{array}{c} 3.\forall i=1,\ldots ,m:{\tilde{y}}_{i,k+1}≔\text{pro}{\text{x}}_{\sigma H_{i}^{*}}(y_{i,k}+\sigma L_{i}(2{\tilde{w}}_{k+1}-w_{k})), \end{array}\\ \begin{array}{c} 4.\forall i=1,\ldots ,m:y_{i,k+1}≔\rho {\tilde{y}}_{i,k+1}+(1-\rho )y_{i,k.} \end{array} \end{array}$$

where for a proper, lower semicontinuous, convex function *G*, prox_*τG*_ is its proximal operator, defined as:

(4.10) $$\text{pro}{\text{x}}_{\tau G}(y)≔\arg\underset{x}{\text{ min}}\{\frac{1}{2\tau }∥x-y{∥}_{2}^{2}+G(x)\}.$$

The iterates ${\left(w_{k}\right)}_{k\in \mathbb{N}}$ and ${\left(y_{i,k}\right)}_{k\in \mathbb{N}}(i=1,\ldots ,m)$ are shown to converge if the parameters *σ* and *τ* are chosen such that $\sigma \tau \|\sum_{i=1}^{m}L_{i}^{*}L_{i}\|\leq 1$ (see [48, Theorem 5.3]). In step 3 in (4.9), we use Moreau’s identity to obtain prox_σH_^*_i_^ from prox_*H*_*i*/*σ*__:

(4.11) $$\text{pro}{\text{x}}_{\sigma H_{i}^{*}}(y)+\sigma \text{pro}{\text{x}}_{H_{i}/\sigma }(y/\sigma )=y.$$

As a stopping criterion, one can use the primal–dual gap, i.e. the difference between the primal objective cost at the current iterate and the dual objective cost at the current (dual) iterate:

(4.12) $$\begin{array}{l} D_{pd}(w,y_{1},\ldots ,y_{m})\\ =G(w)+\sum_{i=1}^{m}H_{i}(L_{i}w)+G^{*}(-\sum_{i=1}^{m}L_{i}^{*}y_{i})+\sum_{i=1}^{m}H_{i}^{*}(y_{i}) \end{array}$$

Due to strong duality, optimality is reached when the primal– dual gap is zero, so a practical stopping criterion is when the gap reaches a certain threshold set in advance.

Lastly, note that the optimisation is performed jointly over both *u* and *v*, which introduces a difficulty for the term *H*_2_(*L*_2_*w*) in Step 3 above, as this requires the proximal operator of the joint Kullback–Leibler divergence *D_KL_(u, v)*. Similarly, the computation of the primal–dual gap in (4.12) requires the convex conjugate of the joint Kullback–Leibler divergence. We describe the details of these computations in Sects. 4.2 and 4.3, respectively.

### 4.2 Computing the Proximal Operator of the Joint Kullback–Leibler Divergence

When writing the optimisation problem in the form (4.2), it is common that the functions *G* and *H_i_* (*i* = 1*, … , m*) are “simple”, meaning that their proximity operators have a closed-form solution or can be easily computed with high precision. This is certainly true for *G* and *H*_1_, but not obvious for the joint Kullback–Leibler divergence.

First, for discrete images *u* = [*u*_1_*, … , u_N_*]^*T*^, [*v*_1_*, … , v_N_*]^*T*^, the definition (3.6) becomes:

(4.13) $$D_{KL}(v,u)=\sum_{j=1}^{N}u_{j}-v_{j}+v_{j}\log\frac{v_{j}}{u_{j}}$$

and then:

(4.14) proxγDKL(u∗,v∗)=arg minu,vDKL(u,v)+12γ‖[uv]−[u∗v∗]‖22=arg minu,v∑j=1Nuj−vj+vjlogvjuj+12γ[(uj−uj∗)2+(vj−vj∗)2]=∑j=1Narg minuj,vjΦ(uj,vj),

where we define the function Φ as:

(4.15) $$\begin{array}{c} \text{Φ}(u_{j},v_{j}):=u_{j}-v_{j}+v_{j}\log\frac{v_{j}}{u_{j}}+\frac{1}{2\gamma }[{\left(u_{j}-u_{j}^{*}\right)}^{2}+{\left(v_{j}-v_{j}^{*}\right)}^{2}]. \end{array}$$

To find the minimiser of Φ *(u_j_, v_j_)*, we let its gradient be equal to zero:

(4.16) $$\begin{array}{c} \{\begin{array}{c} \partial_{u_{j}}\text{Φ}(u_{j},v_{j})=0\\ \partial_{v_{j}}\text{Φ}(u_{j},v_{j})=0 \end{array}\\ \;\Leftrightarrow \{\begin{array}{c} 1-\frac{v_{j}}{u_{j}}+\frac{1}{\gamma }(u_{j}-u_{j}^{*})=0\\ \log v_{j}-\log u_{j}+\frac{1}{\gamma }(v_{j}-v_{j}^{*})=0 \end{array}. \end{array}$$

In the second equation, we write *u_j_* as a function of *v_j_*, which we substitute in the first equation to obtain:

(4.17) $$\{\begin{array}{l} 1-e^{-\frac{1}{\gamma }(v_{j}-v_{j}^{*})}+\frac{1}{\gamma }(v_{j}e^{\frac{1}{\gamma }(v_{j}-v_{j}^{*})}-u_{j}^{*})=0\\ u_{j}=v_{j}e^{\frac{1}{\gamma }(v_{j}-v_{j}^{*})} \end{array}.$$

The first equation is then solved using Newton’s method, where the iteration is given by:

(4.18) $$v_{j}^{\left(k+1\right)}=v_{j}^{\left(k\right)}-\frac{\gamma -\gamma e^{-\frac{1}{\gamma }(v_{j}^{\left(k\right)}-v_{j}^{*})}+v_{j}^{\left(k\right)}e^{\frac{1}{\gamma }(v_{j}^{\left(k\right)}-v_{j}^{*})}-u_{j}^{*}}{e^{-\frac{1}{\gamma }(v_{j}^{\left(k\right)}-v_{j}^{*})}+(1+\frac{1}{\gamma }v_{j}^{\left(k\right)})e^{\frac{1}{\gamma }(v_{j}^{\left(k\right)}-v_{j}^{*})}}.$$

### 4.3 Computing the Convex Conjugate of the Joint Kullback–Leibler Divergence

We compute the convex conjugate of the discrete joint Kullback–Leibler divergence *D_KL_(v, u)* in (4.13) for *u, v* ∈ [*l*_1_*, l*_2_]^*N*^ :

(4.19) $$\begin{array}{lll} D_{KL}^{*}(v^{*},u^{*})=\underset{v,u\in {\left[l_{1},l_{2}\right]}^{N}}{\sup}\langle \left[\begin{array}{l} u\\ v \end{array}],[\begin{array}{l} u^{*}\\ v^{*} \end{array}\right]\rangle -D_{KL}(v,u) & & \\ \begin{array}{c} \;=\underset{v,u\in {\left[l_{1},l_{2}\right]}^{N}}{\sup}\sum_{j=1}^{N}u_{j}u_{j}^{*}+v_{j}v_{j}^{*}\\ \;-u_{j}+v_{j}-v_{j}\log\frac{v_{j}}{u_{j}}\\ \;=\sum_{j=1}^{N}\underset{v_{j},u_{j}\in [l_{1},l_{2}]}{\sup}\text{Ψ}(v_{j},u_{j}), \end{array} & & \end{array}$$

where ψ is defined as:

(4.20) $$\text{Ψ}(v_{j},u_{j}):=u_{j}u_{j}^{*}+v_{j}v_{j}^{*}-u_{j}+v_{j}-v_{j}\log\frac{v_{j}}{u_{j}}.$$

To solve the optimisation problem on the last line in (4.19), we write the KKT conditions (where we use *u, v* instead of *u_j_, v_j_* to simplify the notation:

(4.21a) $$-\nabla \text{Ψ}(v,u)+\sum_{i=1}^{4}\mu_{i}\nabla g_{i}(v,u)=0,$$

(4.21b) $$g_{i}(v,u)\leq 0,\;\forall i=1,\ldots ,4,$$

(4.21c) $$\mu_{i}\geq 0,\;\forall i=1,\ldots ,4,$$

(4.21d) $$\mu_{i}g_{i}(v,u)=0,\;\forall i=1,\ldots ,4.$$

where the functions *g_i_* correspond to the bound constraints:

(4.22a) $$g_{1}(v,u)=u-l_{2};$$

(4.22b) $$g_{2}(v,u)=v-l_{2};$$

(4.22c) $$g_{3}(v,u)=-u+l_{1};$$

(4.22d) $$g_{4}(v,u)=-v+l_{1};$$

Noting that (4.21a) is equivalent to:

(4.23a) $$-u^{*}+1-\frac{v}{u}+\mu_{1}-\mu_{3}=0,$$

(4.23b) $$-v^{*}+\log v-\log u+\mu_{2}-\mu_{4}=0,$$

we solve the last two equations by using the complementarity conditions (4.21d) for cases when the Lagrange multipliers *μ_i_* are zero or nonzero.

## 5 Numerical Results

In this section, we describe a number of numerical experiments that illustrate the performance of our deconvolution method. We start with four examples of simulated data, where we are able to quantify the reconstructed image in relation to the known ground truth image. Then, we show how our method performs on microscopy data, where we reconstruct an image of spherical beads and a sample of a Marchantia thallus. In the experiments with microscopy data, we compare our method with two standard approaches of performing shift-invariant deconvolution, one where the convolution kernel is the detection PSF and one where the convolution kernel is the point-wise multiplication of the detection PSF with the light-sheet.

### 5.1 Simulated Data

**Fig. 8:**
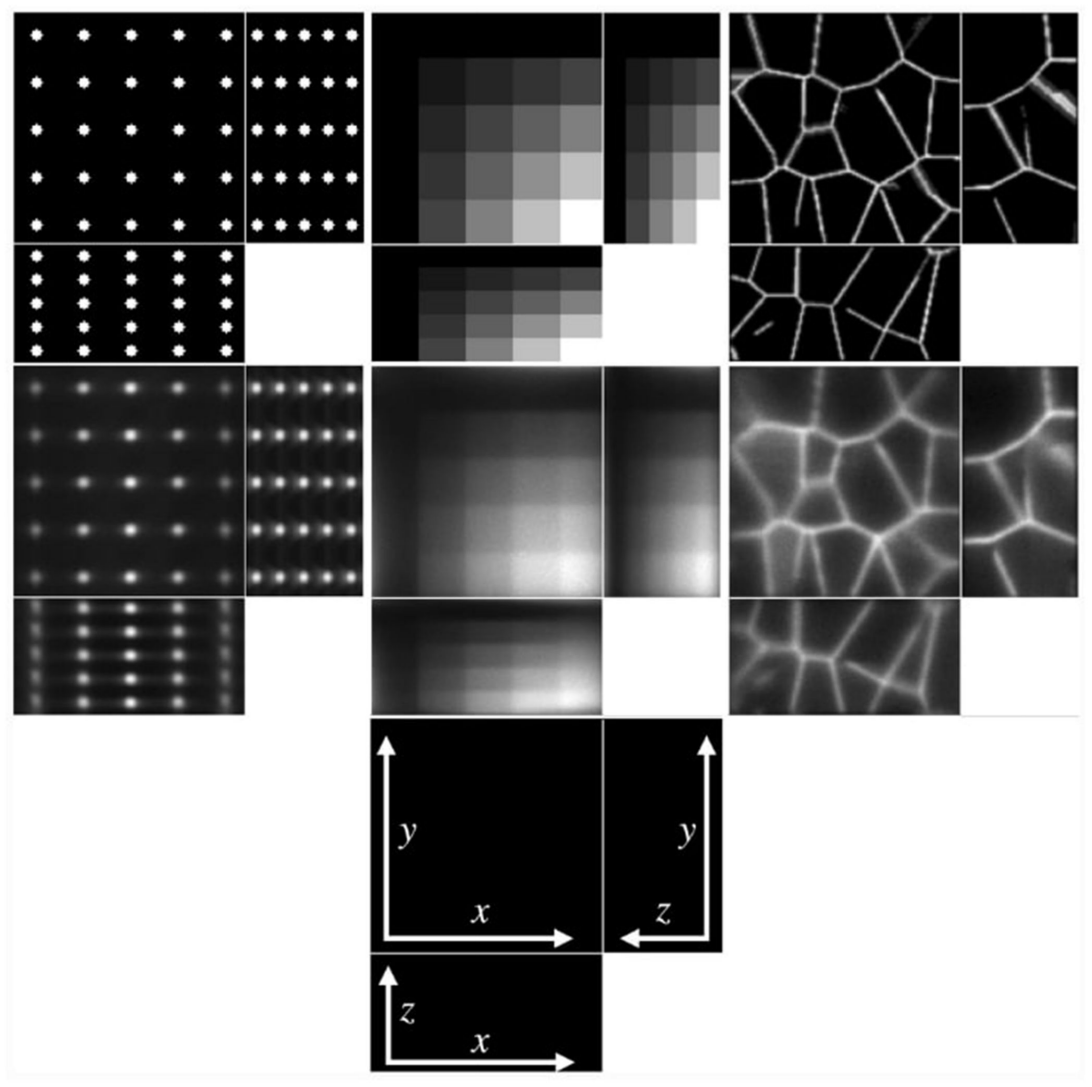
Ground truth (top row) and measured images (middle row), shown using maximum intensity projections in each axis direction, except for the tissue images, where slices in each axis direction are shown. The axes of the plots are shown in the panel in the bottom row, with the missing axis in each panel being the direction in which the maximum intensity projection (or slice) is taken

We consider four images of size 128×125×64: a 5×5×5 grid of beads where the effect of the light-sheet in the *z* coordinate and the shape of the objective PSF are noticeable, a piecewise constant image of “steps” where the Poisson noise affects each step differently based on intensity, and an image that replicates realistic biological samples of tissue. These are shown in the top row of Fig. 8.

To obtain the measured data, we proceed as follows. Given the ground truth image *u*_0_, the forward operator described in Sect. 2.1 is applied to obtain the blurred image *Lu*_0_. The parameters for the forward model are taken to be those of the microscope used in the experimental setup and are given in Table 2. Then, the image is corrupted with a mixture of Poisson and Gaussian noise. For the vectorised image *Lu*_0_, at each pixel *i* = 1*, … , N*, the Poisson noise component follows the Poisson distribution with parameter (*Lu*_0_)*_i_* and the additive Gaussian component has zero mean and standard deviation *σ_G_* = 10. The original image, which has intensity in [0, 1] is scaled so that the intensity of *Lu*_0_ is in [0, 2000], to replicate a realistic scenario for the Poisson noise intensity. The resulting simulated measured data is shown in the bottom row of Fig. 8.

We compare the reconstruction obtained using the proposed approach, which we will refer to as LS-IC (light-sheet-infimal convolution), with the reconstructions obtained by using an *L*^2^ data fidelity term instead of the infimal convolution term, or using a convolution operator corresponding to the objective PSF instead of the light-sheet forward model from Sect. 2.1. Specifically, we compare the solution of (4.1) with the solutions to the following problems, all solved using PDHG as described in Sect. 4:

(PSF-L2) minu αTV(u)+12σG2∥f−Hu∥22+χ[0,B]2N([u,v]T), 

(PSF-IC) minu,v αTV(u)+12σG2∥f−v∥22+DKL(v,Hu)  +χ[0,B]2N([u,v]T),

(LS-L2) minu αTV(u)+12σG2∥f−Lu∥22+χ[0,B]2N([u,v]T), 

where *H* is the convolution operator with the detection objective PSF *h_z_* as given in (2.14).

For each test image and each method above, the PDHG parameters *ρ* and *σ* used are given in Table 3 and *τ* is set to $\tau =1/\sigma \|\sum_{i=1}^{m}L_{i}^{*}L_{i}\|$ to ensure convergence according to Theorem 5.3 in [48]. As a stopping criterion, we used the primal–dual gap (4.12), normalised by the number of pixels *N* and the dynamic range of the measured image *f* :

(5.1) $${\tilde{D}}_{pd}=\frac{D_{pd}}{N\cdot \underset{}{\max_{j=1,\ldots ,N}}f_{j}},$$

with a threshold of 10^−6^ and a maximum number of 10,000 iterations.

**Fig. 9:**
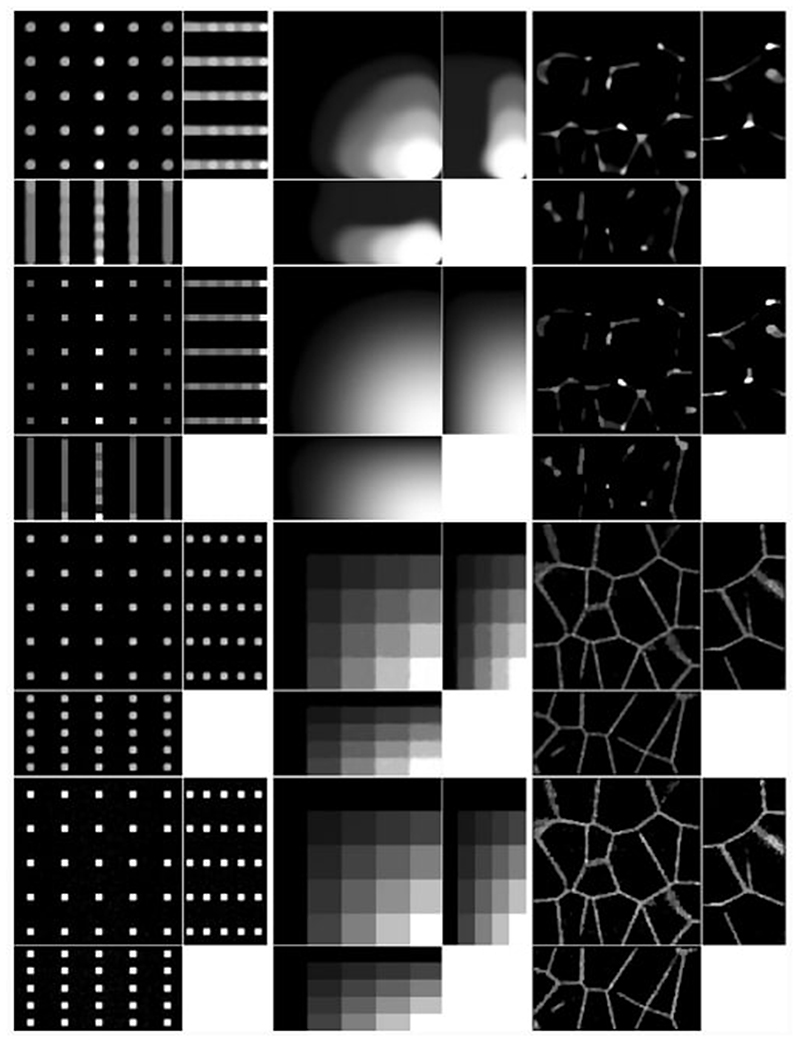
Reconstruction on simulated data with regularisation parameter *α* such that best MSE is achieved for each method and each image. Shown as maximum intensity projections, except for tissue, where slices in each direction in the centre of the sample are shown. The axes are as shown in the bottom row of Fig. 8. *First row*: PSF-L2. *Second row*: PSF-IC. *Third row*: LS-L2. *Fourth row*: LS-IC

The results of the four methods applied to the test images are given in Fig. 9 and quantitative results are given in Table 4. For each test image and each method, the regularisation parameter has been chosen to optimise the normalised *l*^2^ error and the structural similarity index (SSIM), respectively.

**Fig. 10:**
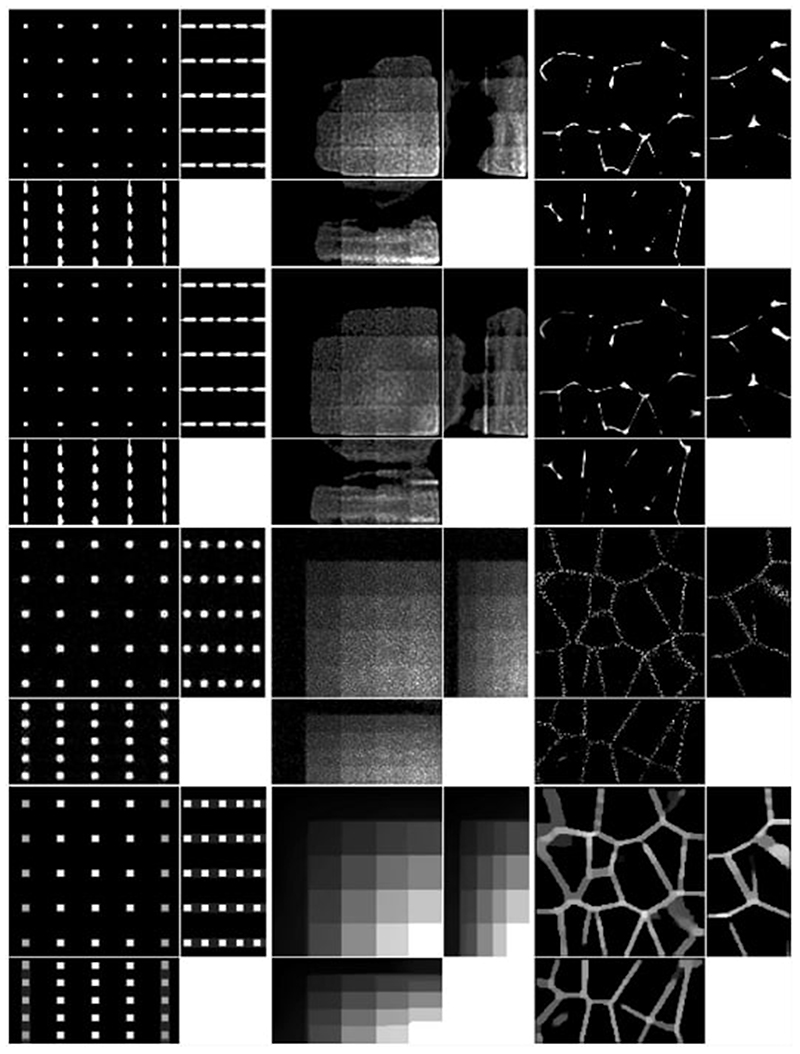
Reconstruction on simulated data with regularisation parameter *α* chosen to satisfy the discrepancy principle (3.11). Shown as maximum intensity projections, except for tissue, where slices in each direction in the centre of the sample are shown. The axes are as shown in the bottom row of Fig. 8. *First row*: PSF-L2. *Second row*: PSF-IC. *Third row*: LS-L2. *Fourth row*: LS-IC

We note that PSF-L2 and PSF-IC perform particularly poorly, highlighting the importance of an accurate representation of the image formation model instead of simply using the detection objective PSF as the forward operator. Comparing LS-IC and LS-L2, we see better results when using the infimal convolution data fidelity for the beads and the steps image, both visually and quantitatively. The deblurring is performed better on the beads image, while on the steps image we see a better denoising effect, especially along the edges in the image. For the tissue image, both fidelities give comparable results, but as we see in Fig. 10, when the ground truth is not known, choosing *α* using the discrepancy principle gives a better result for the infimal convolution model.

The reconstructions shown in Fig. 10 are obtained by applying the discrepancy principle corresponding to each method. For LS-IC, we choose a value of *α* such that it satisfies a variation of the discrepancy principle given in (3.11), where we enforce that the single noise fidelities are bounded by their respective noise bounds, rather than the sum of the fidelities being bounded by the sum of the noise bounds, as stated in (3.11). While both versions give good results, we found the former to give more accurate reconstructions. Here, the bound on the Poisson noise is set to $\frac{1}{2}$, motivated by the following lemma from [49], which gives the expected value of the Kullback–Leibler divergence:

**Lemma 5.1** *Let Y_β_ be a Poisson random variable with expected value β and consider the function:*

$$F(Y_{\beta })=2\{Y_{\beta }\log(\frac{Y_{\beta }}{\beta })+\beta -Y_{\beta }\}.$$

*Then, for large β, the following estimate of the expected value of F(Y_β_) holds:*

$$E[F(Y_{\beta })]=1+O(\frac{1}{\beta }).$$

One last observation worth making about the results in Figs. 9 and 10 is about the square shape of the reconstructed beads (the first column of both figures). By looking carefully at the ground truth bead image in Fig. 8, one can see that the beads are almost square to begin with, due to the small dimensions of the image. The finer details that make them appear round are lost in the blurring process which, in combination with the total variation regulariser used in the deconvolution algorithm, leads to this detail not being present in the reconstruction, thus making them square. Moreover, the sharpening of their edges is an expected effect of the total variation regularisation, which could be avoided by using a different regularisation technique. However, this is beyond the scope of this article.

The experiments were run using Matlab version R2020b Update 2 (9.9.0.1524771) 64-bit in Scientific Linux 7.9 on a machine with Intel Xeon E5-2680 v4 2.40GHz CPU, 256GB memory and Nvidia P100 16GB GPU. The running times, averaged over 5 runs for each method and each image, are given in Table 5.

### 5.2 Light-Sheet Data

In this section, we show the results of applying LS-IC to a cropped portion of the full resolution images in Fig. 2. Specifically, we select a cropped beads image of 1127×111×100 voxels and a cropped Marchantia image of 1127×156× 100 voxels.

For comparison, we also run PSF-L2 on the same images. In addition, we run an alternative light-sheet deconvolution method, where we perform shift-invariant deconvolution using a PSF $\bar{h}$ obtained by point-wise multiplication of the detection PSF *h_z_* in (2.14) and the light-sheet *l*, effectively clipping *h* by the width of the light-sheet. Therefore, the problem we solve, which we denote by PSF-L2-clip is:

(PSF-L2-clip) minu αTV(u)+12σG2∥f−H¯u∥22+χ[0,B]2N([u,v]T), 

where $\bar{H}$ is the convolution operator with the PSF $\bar{h}=h_{z}\cdot l$. A justification of this method is given by a simplified image formation model where we assume that the light-sheet has constant width (in the *z* direction) and constant intensity throughout the full sample, or in a region of interest where deconvolution is performed, as it is done for example in [21].

We run each method on both images for up to 6000 iterations, with a normalised primal–dual gap of 10^−6^ as a stopping criterion. The parameters for the image formation model used are the same as in Table 2 and the PDHG parameters are given in Table 6.

**Fig. 11:**
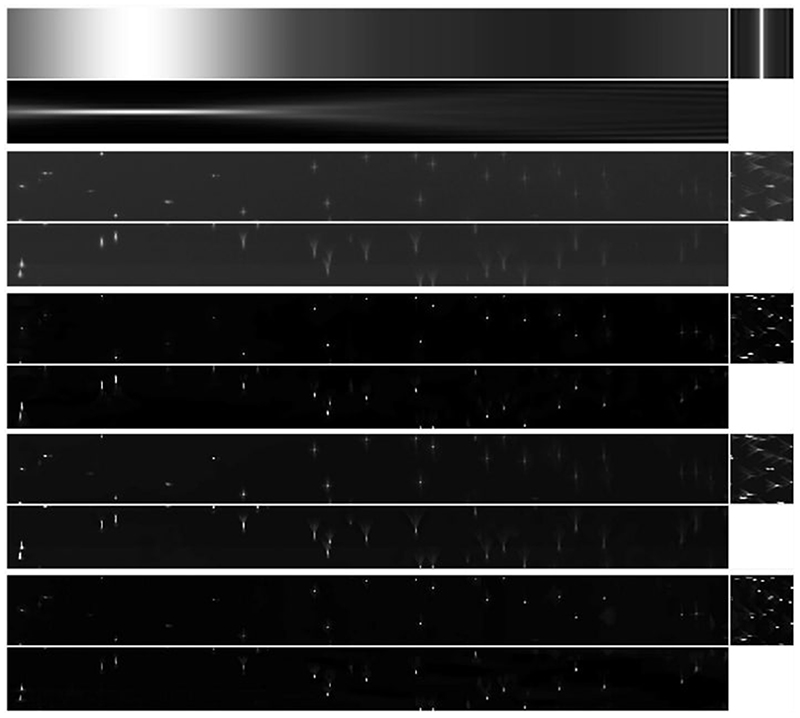
Reconstruction results for the light-sheet bead image, shown as maximum intensity projections. The axes are as shown in the bottom row of Fig. 8. *First row*: The fitted light-sheet profile. *Second row*: The data. *Third row*: PSF-L2 with *α* = 0.1. *Fourth row*: PSF-L2-clip with *α* = 0.7943. *Fifth row*: LS-IC with *α* = 0.0046

The results of the deconvolution are shown in Figs. 11 and 13 for the beads image and the Marchantia image, respectively. In both figures, we first show the position of the light-sheet in the first row (due to the cropping, this is no longer centred), the measured data in the second row, followed by the PSF-L2, the PSF-L2-clip and the LS-IC reconstructions on the third, fourth and fifth rows, respectively. The regularisation parameter *α* was chosen in all four cases visually such that a balance is achieved between the amount of regularisation and the noise in the reconstruction.

**Fig. 12:**
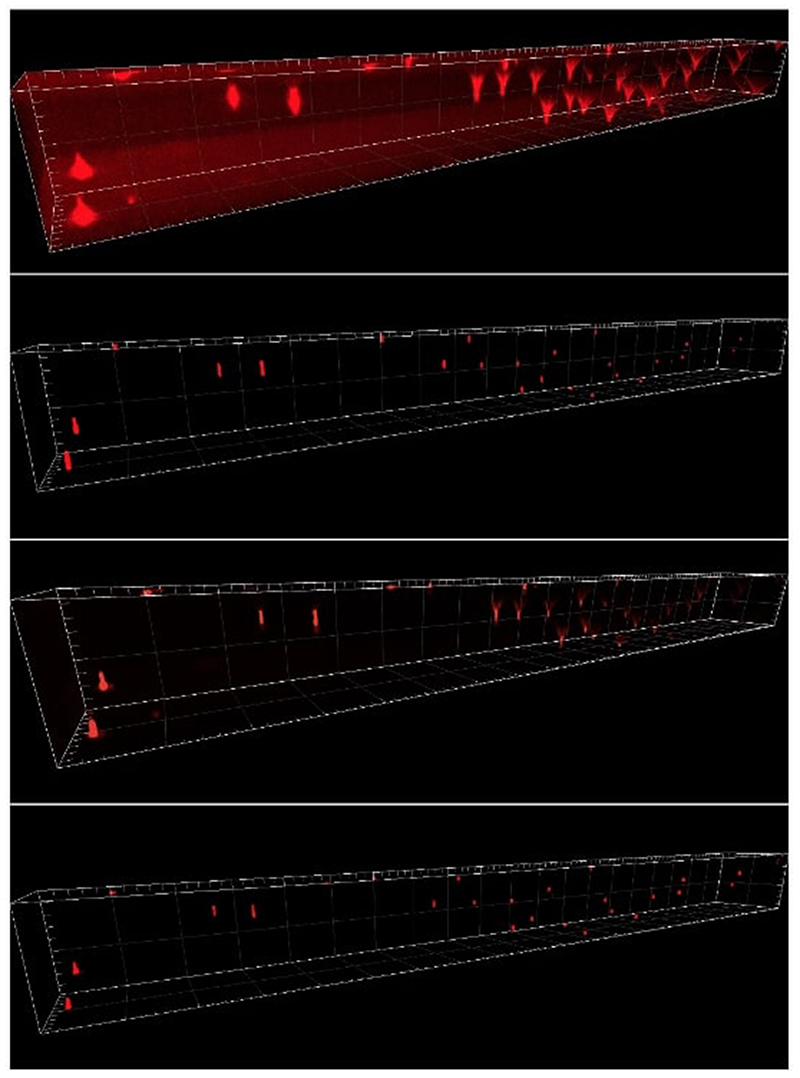
3D rendering of the beads data and reconstruction images using Imaris Viewer 9.7.2. *First row*: The data. *Second row*: PSF-L2 with *α* = 0.1. *Third row*: PSF-L2-clip with *α* = 0.7943. *Fourth row*: LS-IC with *α* = 0.0046

In the beads image in Fig. 11, we note that LS-IC performs better than PSF-L2 and PSF-L2-clip at reversing the effect of the light-sheet. This is most obvious in the *zy* plane on the right-hand side of the image, where the length of the beads in the *z* direction has been reduced to a greater extent than in the PSF-L2 and the PSF-L2-clip reconstructions. In addition, the beads appear less blurry in the LS-IC reconstruction in the right-hand side of the *xy* plane. We also note that PSF-L2-clip fails to properly reverse the effects of the optical aberrations in the beads. This is not unexpected, as the information related to the aberrations is lost when the detection PSF is clipped by setting its upper and lower extremities to zero. The extent to which this happens depends on the width of the light-sheet: as the light-sheet becomes wider, the overall PSF approaches the detection PSF, in which case the deconvolved image will be the same as the reconstruction using PSF-L2. We show the bead images in 3D in Fig. 12, where the effect of the deconvolution in the *z* direction is more significant in the LS-IC reconstruction than in both the PSF-L2 and the PSF-L2-clip reconstructions, namely the beads are shorter in the *z* direction.

**Fig. 13:**
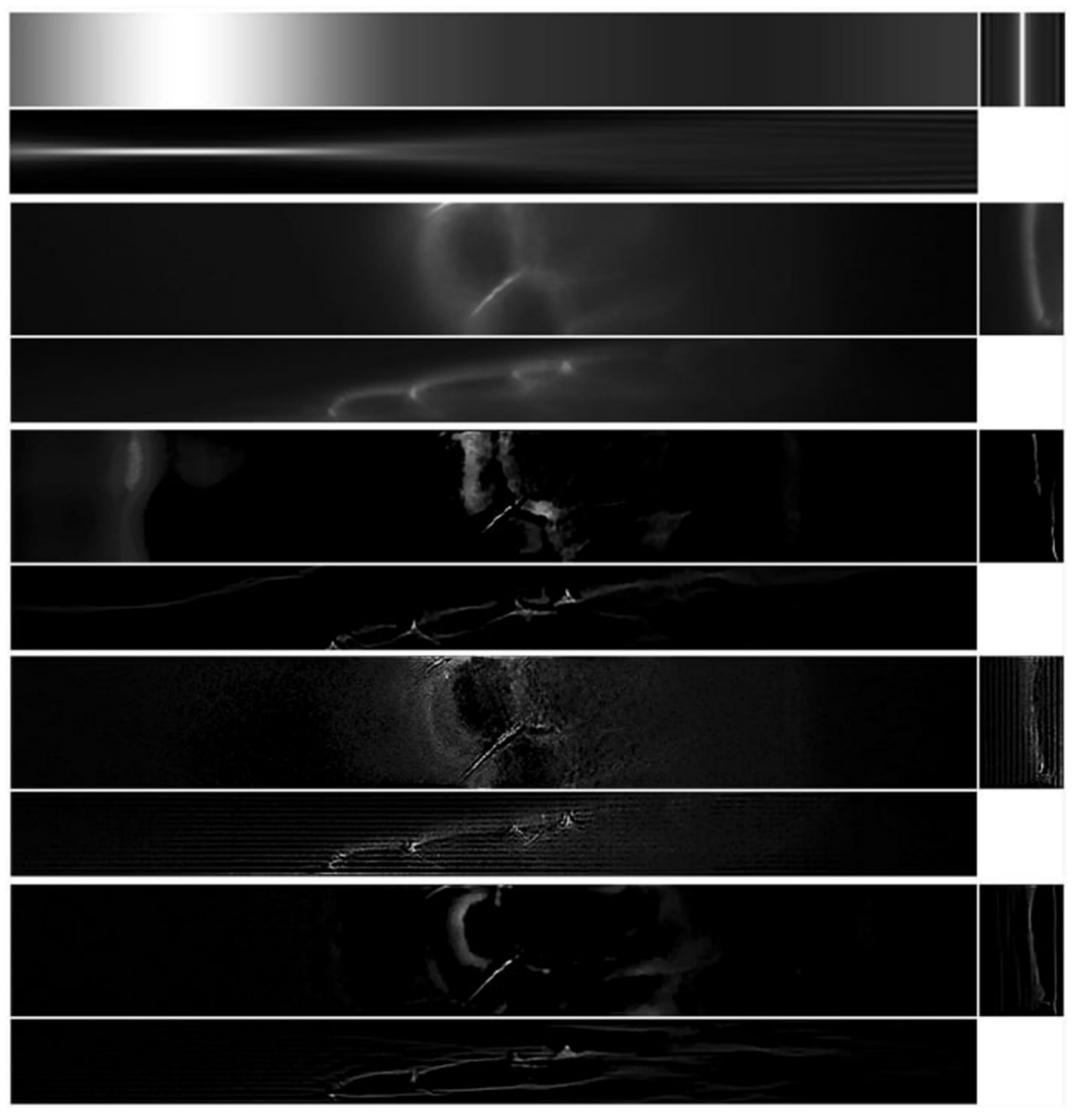
Reconstruction results for the Marchantia sample, shown as slices in each direction in the centre of the sample. The axes are as shown in the bottom row of Fig. 8. *First row*: The fitted light-sheet pro-file. *Second row*: The data. *Third row*: PSF-L2 with *α* = 0.1. *Fourth row*: PSF-L2-clip with *α* = 0.1. *Fifth row*: LS-IC with *α* = 0.0005

**Fig. 14:**
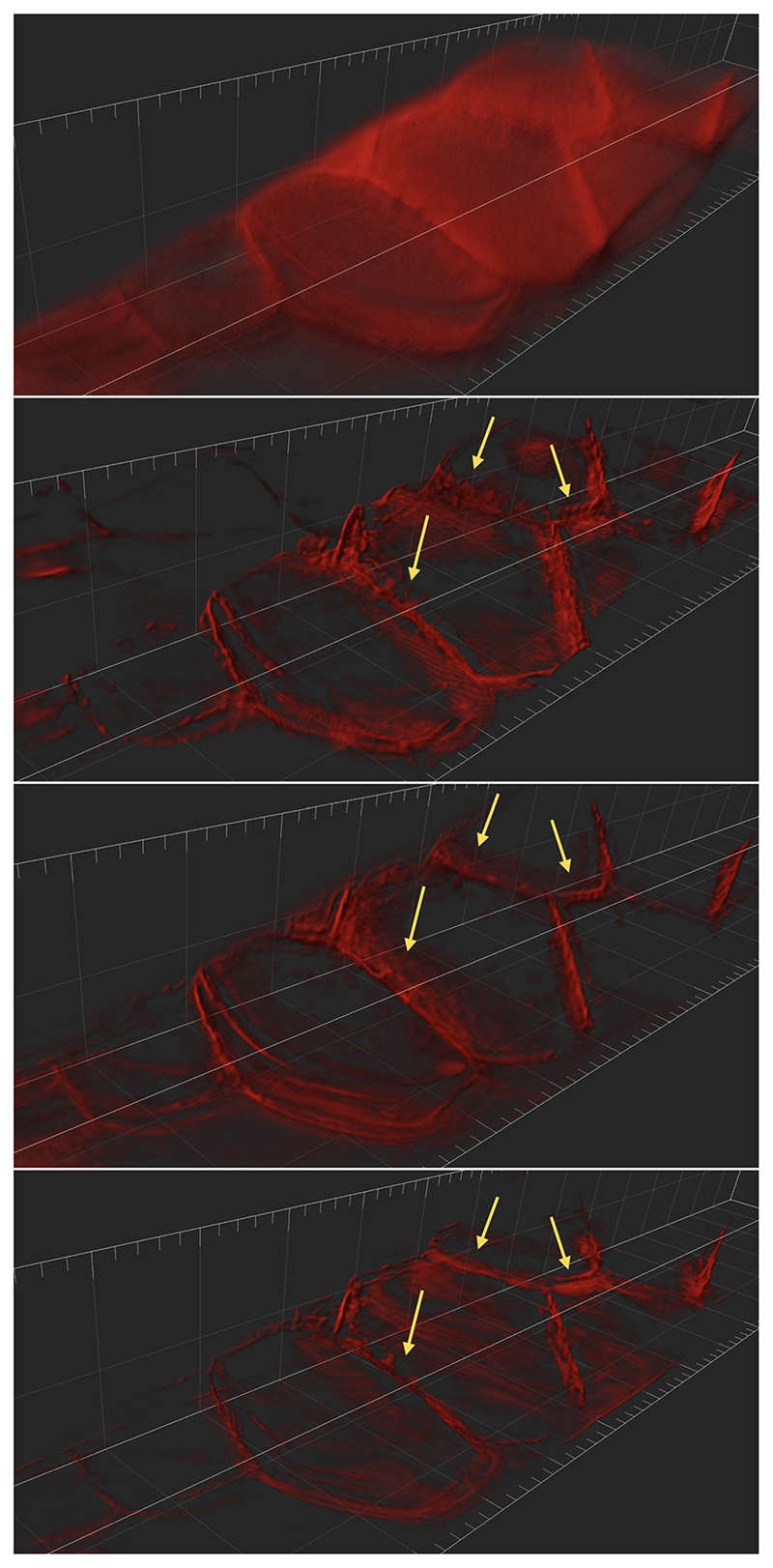
3D rendering of the Marchantia data and reconstruction images using Imaris Viewer 9.7.2. *First row*: The data. *Second row*: PSF-L2 with *α* = 0.1. *Third row*: PSF-L2-clip with *α* = 0.1. *Fourth row*: LS-IC with *α* = 0.0005

In the Marchantia reconstruction in Fig. 13, we see a similar effect of better sharpening in the *z* direction, most easily seen in the right-hand side and bottom projections in each panel (maximum intensity projections in the *zy* and the *xz* planes, respectively). In particular, we see additional artefacts in the PSF-L2-clip reconstruction: horizontal lines (parallel with the *xy* plane), likely due to the clipping of the detection PSF. Moreover, the 3D rendering of the Marchantia sample in Fig. 14 shows smoother cell edges in the LS-IC reconstruction compared to the other methods. Specifically, the PSF-L2 reconstruction contains reconstruction artefacts that are non-existent in the LS-IC reconstruction (indicated by the yellow arrows), while the PSF-L2-clip reconstruction contains areas where the blur has not been fully removed (for example at the same locations indicated by the yellow arrows), where the edges are not as sharp as in the LS-IC reconstruction.

Lastly, we reiterate that the strength of our proposed method is given by the physically accurate modelling of the interaction between the detection PSF and the light-sheet. This allows one to model the optical aberrations as part of the detection PSF (with no requirements on how this should be done), as well as the spatial dependence of the width and the intensity of the light-sheet and to combine them in an image formation model that does not require approximating using a light-sheet with constant width and intensity. As we see in the numerical experiments shown in this section, such approximation, while faster and less expensive computationally, leads to loss of information and results that are at most locally accurate.

## 6 Conclusion

In this paper, we introduced a novel method for performing deconvolution for light-sheet microscopy. We start by modelling the image formation process in a way that replicates the physics of a light-sheet microscope, which is achieved by explicitly modelling the interaction of the illumination light-sheet and the detection objective PSF. Moreover, the optical aberrations in the system are modelled using a linear combination of Zernike polynomials in the pupil function of the detection PSF, fitted to bead data using a least squares procedure. We then formulate a variational model taking into account the image formation model as the forward operator and a combination of Poisson and Gaussian noise in the data. The model combines a total variation regularisation term and a fidelity term that is an infimal convolution between an L^2^ term and the Kullback–Leibler divergence, introduced in [9]. In addition, we establish convergence rates with respect to the noise and we introduce a discrepancy principle for selecting the regularisation parameter *α* in the mixed noise setting. We solve the resulting inverse problem by applying the PDHG algorithm in a non-trivial way.

The results in the numerical experiments section show that our method, LS-IC, outperforms simpler approaches to deconvolution of light-sheet microscopy data, where one does not take into account the variability of the overall PSF introduced by the light-sheet excitation, or the combination of Gaussian and Poisson noise. In particular, numerical experiments with simulated data show superior reconstruction quality in terms of the normalised *l*^2^ error and the structural similarity index, not only by optimising over the regularisation parameter *α* given the ground truth, but also with an a posteriori choice of *α* using the stated discrepancy principle. On bead data, the reconstruction obtained using LS-IC shows a more significant reduction of the blur in the *z* direction compared to PSF-L2, where the light-sheet variations and the Poisson noise are not taken into account. Moreover, reconstruction of a Marchantia sample with LS-IC shows fewer artefacts than the PSF-L2 reconstruction, as well as sharper cell edges and smoother cell membranes.

Future work includes applying this technique to a broader range of samples and using it to answer questions of biological interest. To do so, we see a number of potential future directions that this work can take:

1. Adapting the discrepancy principle given in (3.11) for choosing the regularisation parameter *α* to real data sets, like the ones in Sect. 5.2.
2. Improving the running time of the method potentially by means of randomised approaches.
3. Investigating other regularisation terms.
4. Making the technique available to other users as a more user-friendly tool.

## Figures and Tables

**Table 1:** The first 15 Zernike Polynomials (in polar coordinates) and their coefficients used in *h*_*z*_

*Z_j_*	Polynomial	*c_j_*
*Z*_1_	*ρ* cos *θ*	−0.7763
*Z*_2_	*ρ* sin *θ*	−0.0460
*Z*_3_	2*ρ*^2^ − 1	−2.3608
*Z*_4_	*ρ*^2^ cos 2*θ*	−1.3001
*Z*_5_	*ρ*^2^ sin 2*θ*	0.2024
*Z*_6_	(3*ρ*^2^ − 2*)ρ* cos *θ*	−0.3999
*Z*_7_	(3*ρ*^2^ − 2*)ρ* sin *θ*	0.0348
*Z*_8_	6*ρ*^4^ − 6*ρ*^2^ + 1	−1.2112
*Z*_9_	*ρ*^3^ cos 3*θ*	−0.1521
*Z*_10_	*ρ*^3^ sin 3*θ*	−0.0466
*Z*_11_	(4*ρ*^2^ − 3)*ρ*^2^ cos 2*θ*	−0.0930
*Z*_12_	(4*ρ*^2^ − 3)*ρ*^2^ sin 2*θ*	0.0427
*Z*_13_	(10*ρ*^4^ − 12*ρ*^2^ + 3)*ρ* cos *θ*	−0.0117
*Z*_14_	(10*ρ*^4^ − 12*ρ*^2^ + 3)*ρ* sin *θ*	−0.0581
*Z*_15_	20*ρ*^6^ − 30*ρ*^4^ + 12*ρ*^2^ − 1	−0.0633

**Table 2:** Forward model parameters used in Sect. 5

Parameter	Value	Description, units
*n*	1.35	Refractive index
*NA_h_*	1	Numerical aperture (objective lens)
*NA_l_*	0.25	Numerical aperture (light-sheet)
*λ_h_*	0.525	Wave length (objective lens), μm
*λ_l_*	0.488	Wave length (light-sheet), μm
*px_x_*	0.3250	Pixel size (*x*), μm
*px_y_*	0.3250	Pixel size (*y*), μm
*step_z_*	1	Light-sheet step size (*z*), μm

**Table 3:** Values of the PDHG parameters *ρ* and *σ* used in the numerical experiments with simulated data

Method	LS-IC			LS-L2			PSF-IC			PSF-L2		
Image	Beads	Steps	Tissue	Beads	Steps	Tissue	Beads	Steps	Tissue	Beads	Steps	Tissue
*ρ*	0.9	0.9	0.9	0.9	0.9	0.9	0.9	0.9	0.8	0.9	0.9	0.9
*σ*	0.0001	0.0001	0.00001	0.0001	0.001	0.0001	0.0001	0.0001	0.0001	0.0001	0.001	0.0001

**Table 4:** Results of the numerical experiments on simulated data, with the regularisation parameter *α* chosen to optimise the normalised *l*_2_ error and the SSIM, respectively

Image	Beads		Steps		Tissue	
Error metric	*l*_2_	SSIM	*l*_2_	SSIM	*l*_2_	SSIM
PSF-L2	1.74	0.845	0.499	0.561	1.57	0.592
PSF-IC	1.54	0.844	0.324	0.659	1.65	0.582
LS-L2	0.282	0.982	0.055	0.971	0.301	0.951
LS-IC	0.258	0.983	0.012	0.998	0.349	0.931

**Table 5:** Running times for each method and each simulated test image, averaged over 5 runs, in seconds

Image	Beads	Steps	Tissue
PSF-L2	233	1793	903
PSF-IC	689	1077	1805
LS-L2	2913	2194	2273
LS-IC	972	601	850

The minimisation is stopped when the primal–dual gap is lower than 10^−6^ or the maximum number of 10,000 iterations is reached

**Table 6:** Values of the PDHG parameters *ρ* and *σ* used in the numerical experiments with real data

Method	LS-IC		PSF-L2		PSF-L2-clip	
Image	Beads	Marchantia	Beads	Marchantia	Beads	Marchantia
*ρ*	0.5	0.7	0.9	0.9	0.9	0.9
*σ*	0.0001	0.0001	0.01	0.001	0.01	0.001

## Declarations

1 There is a geometric interpretation of the infimal convolution. Given two functions *φ*_1_, *φ*_2_ on Ω, the epigraph (the set of all points lying on or above the graph of a function) of their infimal convolution *φ*_1_□*φ*_2_ is the sum of the epigraphs of *φ*_1_ and *φ*_2_. This can be easily seen if we rewrite the definition (3.4) as (*φ*_1_□*φ*_2_)(*f*) = inf {*φ*_1_(*u*) + *φ*_2_(*v*)|*u*, *v* ∈ Ω, *u* + *v* = *f* }.
